# Substance P modulates bone remodeling properties of murine osteoblasts and osteoclasts

**DOI:** 10.1038/s41598-018-27432-y

**Published:** 2018-06-15

**Authors:** Tanja Niedermair, Stephan Schirner, Raphael Seebröker, Rainer H. Straub, Susanne Grässel

**Affiliations:** 1 Department of Orthopaedic Surgery, University of Regensburg, Regensburg, Germany; 2 Department of Orthopaedic Surgery, Experimental Orthopaedics, Centre for Medical Biotechnology, University of Regensburg, Regensburg, Germany; 3 Department of Internal Medicine I, Experimental Rheumatology and Neuroendocrine-Immunology, University of Regensburg, Regensburg, Germany

## Abstract

Clinical observations suggest neuronal control of bone remodeling. Sensory nerve fibers innervating bone, bone marrow and periosteum signal via neurotransmitters including substance P (SP). In previous studies we observed impaired biomechanical and structural bone parameters in tachykinin (Tac) 1-deficient mice lacking SP. Here, we aim to specify effects of SP on metabolic parameters of bone marrow macrophage (BMM)/osteoclast cultures and osteoblasts isolated from Tac1-deficient and wildtype (WT) mice. We demonstrated endogenous SP production and secretion in WT bone cells. Absence of SP reduced bone resorption rate, as we found reduced numbers of precursor cells (BMM) and multinucleated osteoclasts and measured reduced cathepsin K activity in Tac1−/− BMM/osteoclast cultures. However, this might partly be compensated by reduced apoptosis rate and increased fusion potential of Tac1−/− precursor cells to enlarged “super” osteoclasts. Contrarily, increased ALP enzyme activity and apoptosis rate during early osteoblast differentiation accelerated osteogenesis and cell death in the absence of SP together with reduced ALP activity of Tac1−/− osteoblasts during late osteogenic differentiation resulting in reduced bone formation at later stages. Therefore, we suggest that absence of SP presumably results in a slight reduction of bone resorption rate but concomitantly in a critical reduction of bone formation and mineralization rate.

## Introduction

Bone needs to be a highly dynamic tissue to assure lifelong adaption to biomechanical forces, stress response and repair of traumata induced damages. Starting during skeletogenesis, bone formation and bone resorption continue in adult organism nearly identical during the process of bone remodeling and fracture healing to preserve skeletal shape and integrity. In a coupled process, controlled by a variety of biochemical and mechanical factors, bone degrading osteoclasts, matrix building osteoblasts as well as osteocytes and lining cells at the bone surface ensure the balance between bone resorption and bone formation. Nevertheless, aging or pathological conditions can change the balance^1–3^.

Bone matrix is remodeled simultaneously at different skeletal sites starting with initial recruitment of hematopoietic myelomonocytic precursor cells and their proliferation within the macrophage lineage. Osteoclastogenesis proceeds via an early osteoclast precursor stage, characterized by the expression of tartrate resistant acid phosphatase (TRAP), followed by cell fusion and differentiation to multinucleated osteoclasts. The essential force driving differentiation to mature bone resorbing osteoclasts is the activation of receptor activator of NFκB (RANK) by binding of RANK ligand (RANKL), which is released by stromal cells and osteoblasts. Finally, the active osteoclast creates an isolated extracellular microenvironment to demineralize bone matrix by acidification and to degrade it via lysosomal protease cathepsin K^4–8^.

Bone resorption is followed by the recruitment of mesenchymal progenitor cells and their maturation to bone forming osteoblasts. During differentiation, these cells express the critical osteoblast markers Runt-related transcription factor 2 (Runx2) and osterix^9–11^. Mature osteoblasts then synthesize matrix proteins as collagen type I, the main component of organic bone matrix, non-collagenous proteins as osteocalcin and the key enzyme alkaline phosphatase (ALP), which is crucial for matrix mineralization. Finally, in a process involving ALP enzyme activity, hydroxyapatite crystals (HA) are incorporated into the newly formed bone matrix to terminate the remodeling cycle^12,13^.

There is growing evidence that the sensory nervous system is one of the factors critically involved in bone cell differentiation, bone metabolism and remodeling. Changes in nerve fiber distribution, profiles and density have been reported in musculoskeletal pathophysiologies^14^. Bone, bone marrow and periosteum are innervated by sensory nerve fibers containing sensory neurotransmitters substance P (SP) and alpha-calcitonin gene-related peptide (a-CGRP)^15,16^. SP, an undeca-amino acid neuropeptide belongs to the tachykinin family and signals peripherally predominantly via the neurokinin 1 receptor (NK1R) detected on several different non-neuronal cell types. Dose-dependently SP stimulates osteoblast precursor proliferation and enhanced cell activity and bone formation in differentiating osteoblasts *in vitro*. Additionally, stimulation with SP facilitated osteoclastogenesis of isolated bone marrow macrophages and bone resorption activity of mature osteoclasts^14,17–19^.

Recently, we reported alterations in osteoclast and osteoblast numbers during fracture callus maturation in SP-deficient (Tachykinin 1 = Tac1 − knockout) mice compared to wildtype (WT)^20^. In addition, we observed impaired bone microarchitecture in the fractured and moreover in the contralateral non-fractured femora of SP-deficient mice indicating changes in bone metabolism and remodeling independent of trauma when SP is missing. These alterations in bone remodeling imply a strong and critical neuro-osteogenic connection. So far, the direct effects of SP on bone cell metabolism are still incompletely understood. We hypothesize that SP dose-dependently affects metabolism and gene expression of osteoblasts and osteoclasts critically. Therefore, the aim of this study was to investigate effects of SP deficiency on metabolic parameters of osteoblasts and macrophages/osteoclasts isolated from SP-deficient mice.

## Results

### Determination of bone marrow macrophage (BMM) and osteoblast numbers

**Figure 1:**
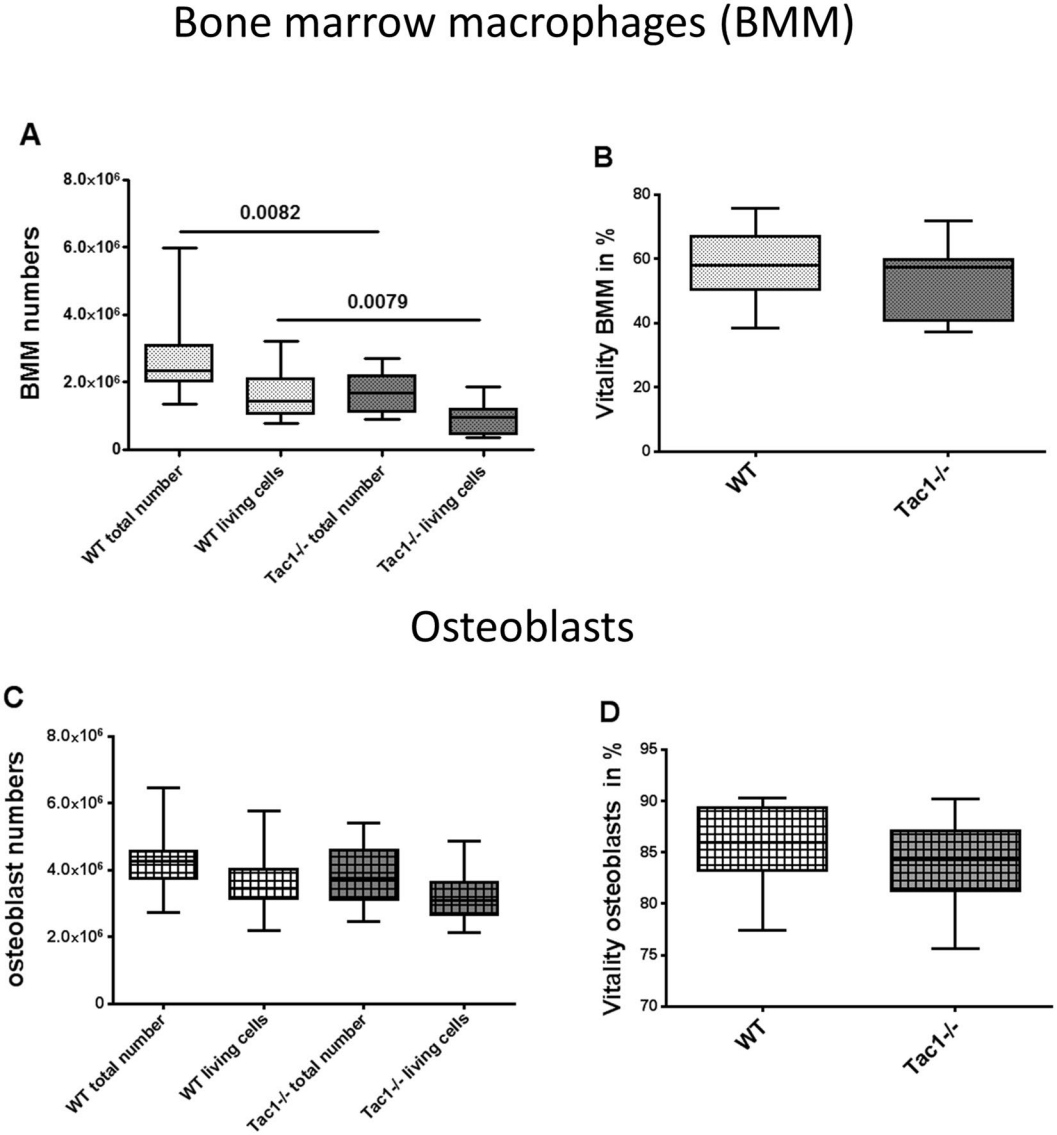
Cell number (**A,C**) and vitality (**B,D**) of BMM (**A,B**) and osteoblasts (**C,D**). Cells were analysed after isolation from bone marrow and outgrowth from bone explants obtained from WT and Tac1−/− mice. N = 12–19.

BMM were isolated from bone marrow of WT and Tac1−/− mice and pre-cultured for 2 days in the presence of M-CSF (20 ng/ml), before cells were harvested and cell number was determined. We observed a significantly lower total cell number and a lower number of living cells isolated from bone marrow of Tac1−/− mice compared to WT (Fig. 1A). No difference in BMM vitality was detected (Fig. 1B). To determine osteoblast-like cell numbers, diaphyseal bone explants prepared from WT and Tac1−/− mice were cultured in growth medium for 14 ± 2 days, before cells were harvested and cell number was analyzed. Number of total and living osteoblast-like cells was similar for WT and Tac1−/− animals (Fig. 1C) as was vitality (Fig. 1D). Likewise, we detected no significant differences after 5, 8 and 12 days culture in growth medium (Supplementary file 1B).

### Neuropeptide production in WT bone cells and serum of WT and Tac1−/− mice

**Figure 2:**
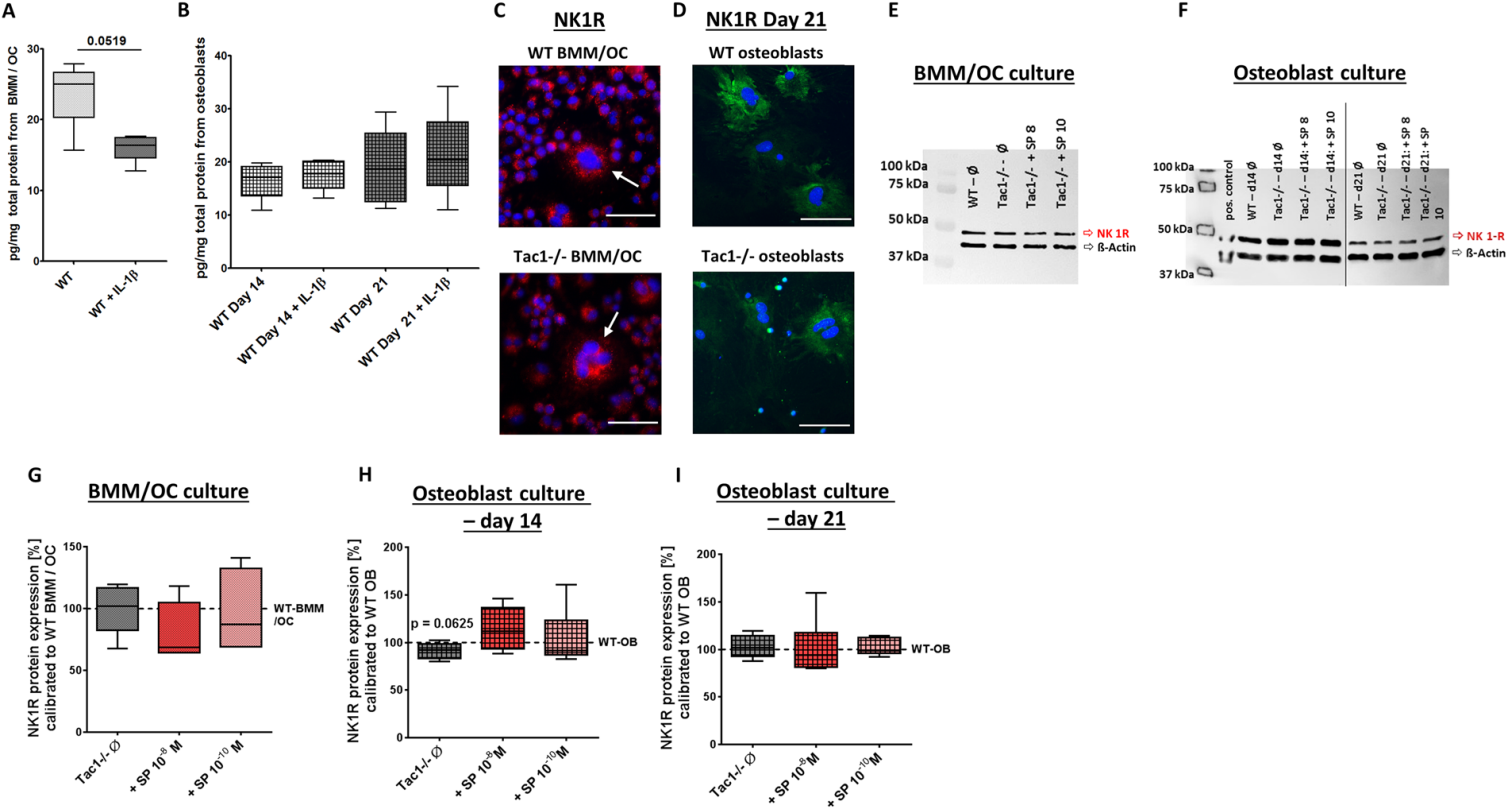
Endogenous SP production and NK1R protein expression. SP concentration in cell culture supernatants of BMM/osteoclast cultures (**A**) and osteoblasts after 14 and 21 days of osteogenic differentiation (**B**), kept for 24 hours in differentiation medium; w/o stimulation with IL-1β (0.5 ng/ml) to mimic proinflammatory conditions. N = 5–8. Assay Detection Limit: 9.76 pg/ml. Endogenous SP (Molecular weight = 1350 g/L) production in BMM/osteoclast cultures and osteoblasts corresponds to about 1–2.5 × 10^−11^ M. (**C**) Representative fluorescence image of NK1R staining (red fluorescence) in WT and Tac1−/− BMM/osteoclast cultures. Size bar = 50 µm. (**D**) Representative fluorescence image of NK1R staining (green fluorescence) in WT and Tac1−/− osteoblasts after 21 days osteogenic differentiation. Size bar = 50 µm Cell nuclei were counterstained with DAPI. (**E**) Representative Western Blot image of NK1R- and β-actin protein expression in WT and Tac1−/− BMM/osteoclast cell lysates, w/o stimulation with 10^−8^/10^−10^ M SP for 24 h. Image is cropped, full length images are provided in Supplementary file 4A. Ø = no stimulation. N = 4–8. (**F**) Representative Western Blot image of NK1R and β-actin protein expression in WT and Tac1−/− osteoblast cell lysates after 14 and 21 days of osteogenic differentiation, w/o stimulation with 10^−8^/10^−10^ M SP for 24 h. Image is cropped from two blots (black line), full length images are provided in Supplementary file 4B (osteoblasts after 14 days) and 4 C (osteoblasts after 21 days). Ø = no stimulation. N = 4–8. Quantification of NK1R protein expression of WT and Tac1−/− BMM/osteoclast cultures (**G**) and osteoblasts after 14 (**H**) and 21 days (**I**) in osteogenic medium, w/o stimulation with 10^−8^/10^−10^ M SP for 24 h, normalized to β-actin protein content. NK1R protein expression in Tac1−/− BMM/osteoclast cultures and osteoblasts was calibrated to WT controls (dotted line = 100%). Ø = no stimulation. N = 4–8. OC = osteoclasts; OB = osteoblasts; *p < 0.05.

SP neuropeptide production was analyzed in WT bone cells during 24 hours of culture time. BMM/osteoclast cultures were differentiated for 5 days and during the last 24 hours of culture time (from day 4 to 5) supernatants of BMM/osteoclast cultures were collected. Analysis revealed that BMM/osteoclast cultures of WT mice produced and secreted SP, corresponding to a mean value of 23.6 (±4.4) pg/mg total protein content. Simulation of inflammatory condition by stimulation of WT-BMM/osteoclast cultures with IL-1β (0.5 ng/ml) significantly reduced SP secretion to a mean of 16.1 (±1.95) pg/mg total protein content (Fig. 2A).

Osteoblast-like cells were differentiated to matrix building osteoblasts for 14 and 21 days in osteogenic medium. Concentration of SP in supernatants of the last 24 hours of culture time (from days 13 to 14 and from 20 to 21) was analyzed (Fig. 2B). At both time points (14/21 days) osteoblasts secreted a mean of 16.4 (±3.3) pg SP/mg total protein, respective 18.9 ± 7.1 pg SP/mg total protein. Stimulation with IL-1β (+0.5 ng/ml IL-1β) had no significant influence on SP concentration (17.4 ± 2.9 pg/mg total protein, respective, 21.3 ± 8.3 pg/mg total protein) in supernatants.

Additionally, we analyzed SP neuropeptide concentration in serum of WT and Tac1−/− mice (Supplementary file 1A). We determined neuropeptide concentrations of 1227 (±837) pg/ml in WT serum and 286.8 (±342.8) pg/ml in serum of Tac1−/− mice.

### NK1R protein expression in WT and Tac1−/− bone cells

Immunofluorescence staining of WT and Tac1−/− BMM/osteoclast cultures (5 days culture) and osteoblasts (21 days culture) revealed NK1R expression (Fig. 2C and D). NK1R protein expression in WT- and Tac1−/− BMM/osteoclast cultures and osteoblasts was quantified and compared (Fig. 2E and F show representative WB images). No differences in NK1R protein expression between WT and Tac1−/− BMM/osteoclast cultures were detected. Stimulation of Tac1−/− BMM/osteoclast cultures with 10^−8^/10^−10^ M SP did not affect NK1R protein expression in these cells compared to the WT controls (Fig. 2G). After 14 days in osteogenic medium, NK1R protein expression was reduced by trend in Tac1−/− osteoblasts with no significant changes after stimulation of Tac1−/− osteoblasts with 10^−8^/10^−10^ M SP (Fig. 2H). After 21 days, NK1R protein expression in Tac1−/− osteoblasts, w/o 10^−8^/10^−10^ M SP was comparable to WT osteoblasts (Fig. 2I).

### Bone cell spreading and growth

**Figure 3:**
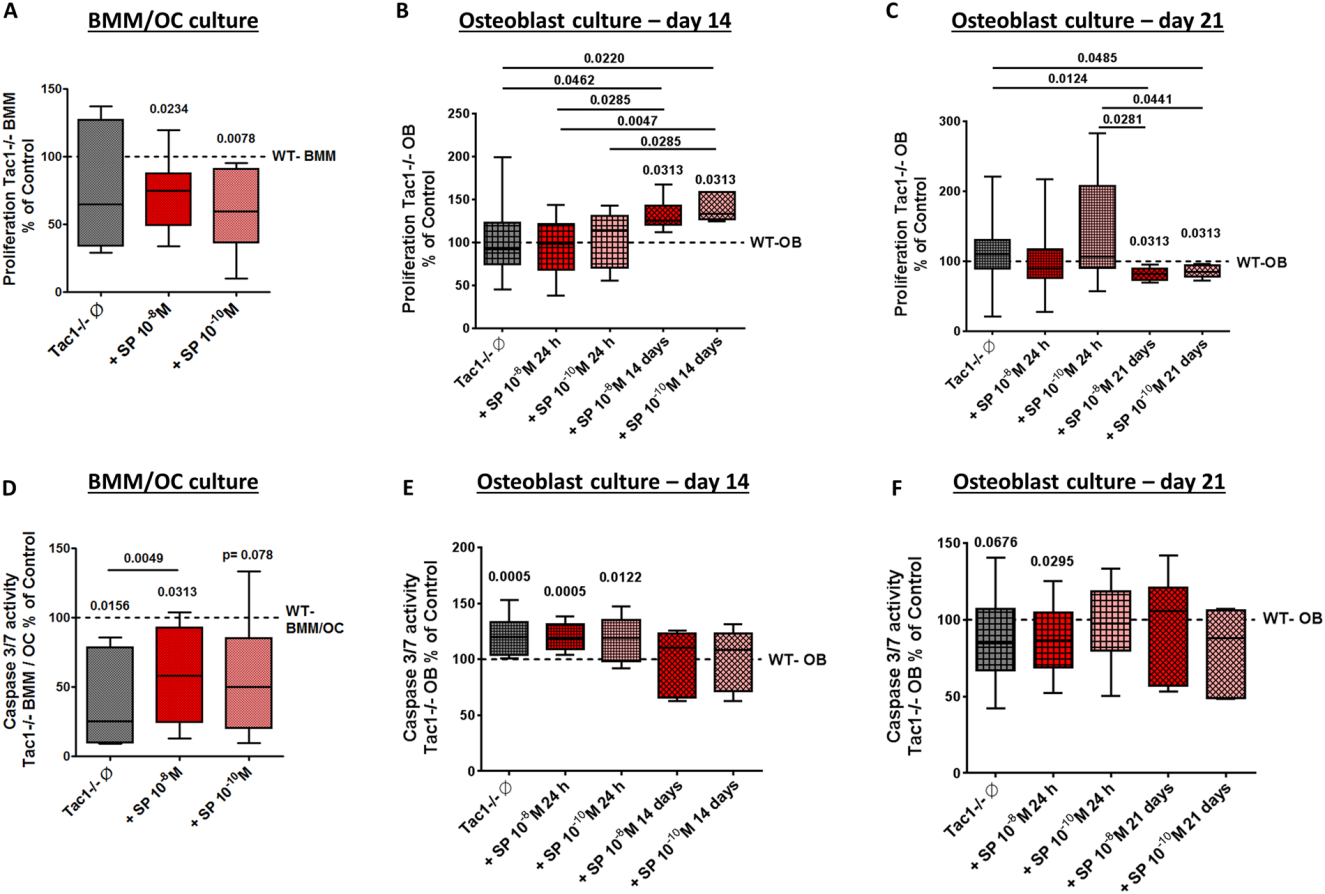
Apoptosis and proliferation rate of BMM/osteoclast and osteoblast cultures. Proliferation of BMM cultures (**A**) and osteoblasts after 14 (**B)** and 21 (**C**) days in osteogenic medium isolated from WT and Tac1−/− mice. N = 8. Caspase 3/7 activity of BMM/osteoclast cultures (**D**) and osteoblasts after 14 (**E**) and 21 (**F**) days in osteogenic medium isolated from WT and Tac1−/− mice. N = 8. BMM/osteoclast cultures and osteoblasts from Tac1−/− mice were stimulated w/o SP 10^−8^/10^−10^ M either for the last 24 h or for the whole culture time (only osteoblast cultures; 14/21 days). Values of WT bone cells were set to 100% and results of Tac1−/− bone cells were calibrated to WT controls (dotted lines = 100%). Ø = no stimulation.

To analyze BMM or osteoblast-like cell spreading behavior, we conducted crystal violet staining 4 hours after seeding the cells. No differences between cell spreading of BMM or osteoblast-like cells from WT controls in comparison to Tac1−/−mice were detected. Stimulation of BMM or osteoblast-like cells isolated from Tac1−/− mice with 10^−8^/10^−10^ M SP did not affect cell spreading (Supplementary file 1B,C). Subsequently, we compared proliferation of BMM isolated from WT and Tac1−/− animals, w/o stimulation with 10^−8^/10^−10^ M SP, after BrdU incorporation for 24 hours. No statistical differences in BrdU incorporation between WT BMM and Tac1−/− BMM were observed. However, after stimulation of Tac1−/− BMM with both concentration of SP, proliferation decreased significantly compared to WT controls (Fig. 3A).

Proliferation of native, unstimulated Tac1−/− osteoblasts after 14 days did not differ from WT osteoblasts in osteogenic medium and similar results were obtained after short-term (24 h) stimulation with 10^−8^/10^−10^ M SP. However, long-term stimulation with 10^−8^/10^−10^ M SP clearly increased BrdU incorporation into Tac1−/− osteoblasts (Fig. 3B) compared to WT or Tac1−/− osteoblasts w/o 24 h stimulation. After 21 days culture in osteogenic medium, no difference in proliferation of WT and Tac1−/− osteoblasts w/o short-term stimulation with 10^−8^/10^−10^ M SP was detected. Long-term stimulation of Tac1−/− osteoblasts with both concentrations of SP decreased cell proliferation compared to WT and native or stimulated (10^−10^ M SP) Tac1−/− osteoblasts (Fig. 3C).

### Determination of caspase 3/7 activity in bone cells

To determine bone cell apoptosis rate we quantified caspase 3/7 activity during the last 24 hours of culture time. Compared to WT, caspase 3/7 activity was strongly reduced in Tac1−/− BMM/osteoclast cultures. Stimulation with 10^−8^ M SP increased caspase 3/7 activity in Tac1−/− BMM/osteoclast cultures compared to native Tac1−/− BMM/osteoclast cultures but did not rescue activity up to WT level (Fig. 3D).

After 14 days culture in osteogenic medium, caspase 3/7 activity was significantly higher in Tac1−/− osteoblasts compared to WT. Short-term stimulation (24 h; 10^−8^/10^−10^ M) of Tac1−/− osteoblasts with SP was without further effect whereas long-term stimulation for 14 days resulted in Caspase 3/7 activity similar to WT level (Fig. 3E). After 21 days culture, caspase 3/7 activity of Tac1−/− osteoblasts was by trend lower compared to WT cells. Short-term stimulation (24 h) with 10^−8^ M SP significantly reduced Caspase 3/7 activity in Tac1−/− osteoblasts compared to WT cells whereas short-term stimulation with 10^−10^ M and long-term stimulation of Tac1−/− osteoblasts had no effects (Fig. 3F) compared to WT or native Tac1−/− osteoblasts.

### Determination of osteoclast numbers, differentiation capacity and resorption activity

**Figure 4:**
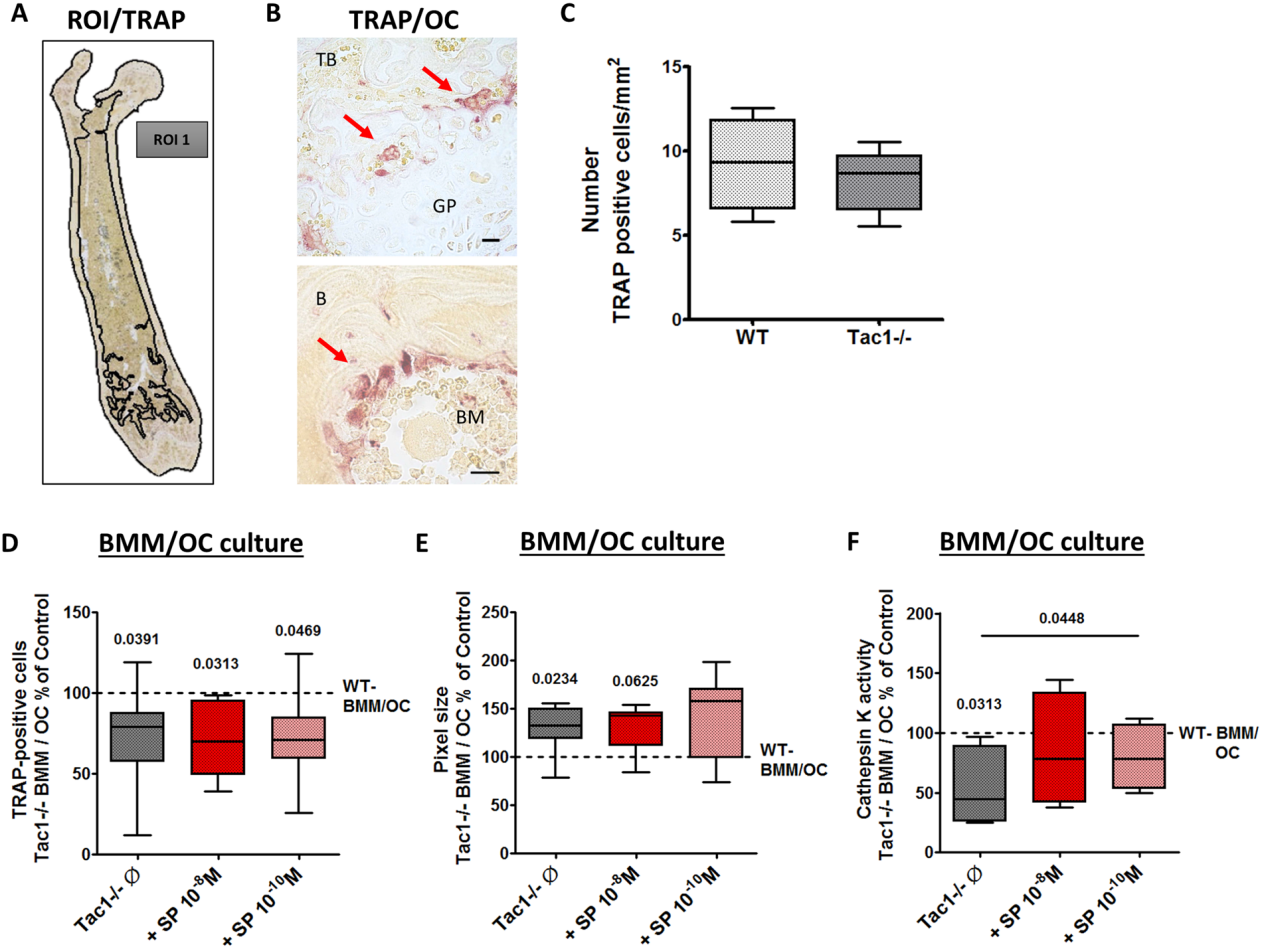
Immunohistological detection of TRAP-positive cells on paraffin sections of WT and Tac1−/− femora and *in vitro* differentiation capacity and activity of BMM/osteoclast cultures. (**A**) Representative image of the region of interest (ROI) set in TRAP stained sections and used to count TRAP-positive cells. (**B**) Representative images of TRAP-positive osteoclasts in paraffin sections of WT femora. Scale bar = 20 µm. N = 4. TB = Trabecular bone; GP = Growth plate; BM = Bone marrow; B = Bone. (**C**) Number of TRAP-positive cells/mm^2^ counted in ROI of femoral paraffin sections of WT and Tac1−/− animals. N = 4. Number (**D**) and pixel area (number of pixel) (**E**) of TRAP positive osteoclasts (≥3 nuclei) after 5 days of M-CSF and RANKL mediated differentiation under cell culture conditions, w/o stimulation of Tac1−/− BMM/osteoclast cultures with SP 10^−8^/10^−10^ M for the last 24 h of culture time. Results of Tac1−/− bone cells were calibrated to WT controls (dotted line = 100%). N = 7–8. (**F**) Cathepsin K enzyme activity after 5 days of differentiation, w/o stimulation of Tac1−/− BMM/osteoclast cultures with SP 10^−8^/10^−10^ M for the last 24 h of culture time. Results of Tac1−/− bone cells were calibrated to WT controls (dotted line = 100%). N = 5–8. Ø = no stimulation.

To determine osteoclast numbers *in vivo*, we analyzed TRAP stained femoral paraffin sections of WT and Tac1−/− mice (representative images in Fig. 4A and B). We did not detect differences in numbers of TRAP-positive cells (Fig. 4C).

BMM isolated of WT and Tac1−/− mice were differentiated *in vitro* to multinucleated osteoclasts. Osteoclast number was counted and cell size was determined by bioimaging (number of pixel). Number of multinucleated osteoclasts was significantly reduced in Tac1−/− BMM/osteoclast cultures and stimulation of Tac1−/− BMM/osteoclast cultures with 10^−8^/10^−10^ M SP had no effects (Fig. 4D). Native Tac1−/− osteoclasts were larger compared to WT osteoclasts and stimulation with 10^−8^/10^−10^ M SP had no significant effect on Tac1−/− osteoclast size (Fig. 4E).

Analysis of cathepsin K activity as a marker for bone resorption activity revealed significantly lower activity in native Tac1−/− osteoclasts compared to WT controls. Stimulation with both SP concentrations resulted in an increase of cathepsin K activity up to WT level and with 10^−10^ M SP resulting additionally in a significant activity difference to native Tac1−/− osteoclasts (Fig. 4F).

### Determination of number of Runx2-positive cells, bone formation activity and calcium deposition

**Figure 5:**
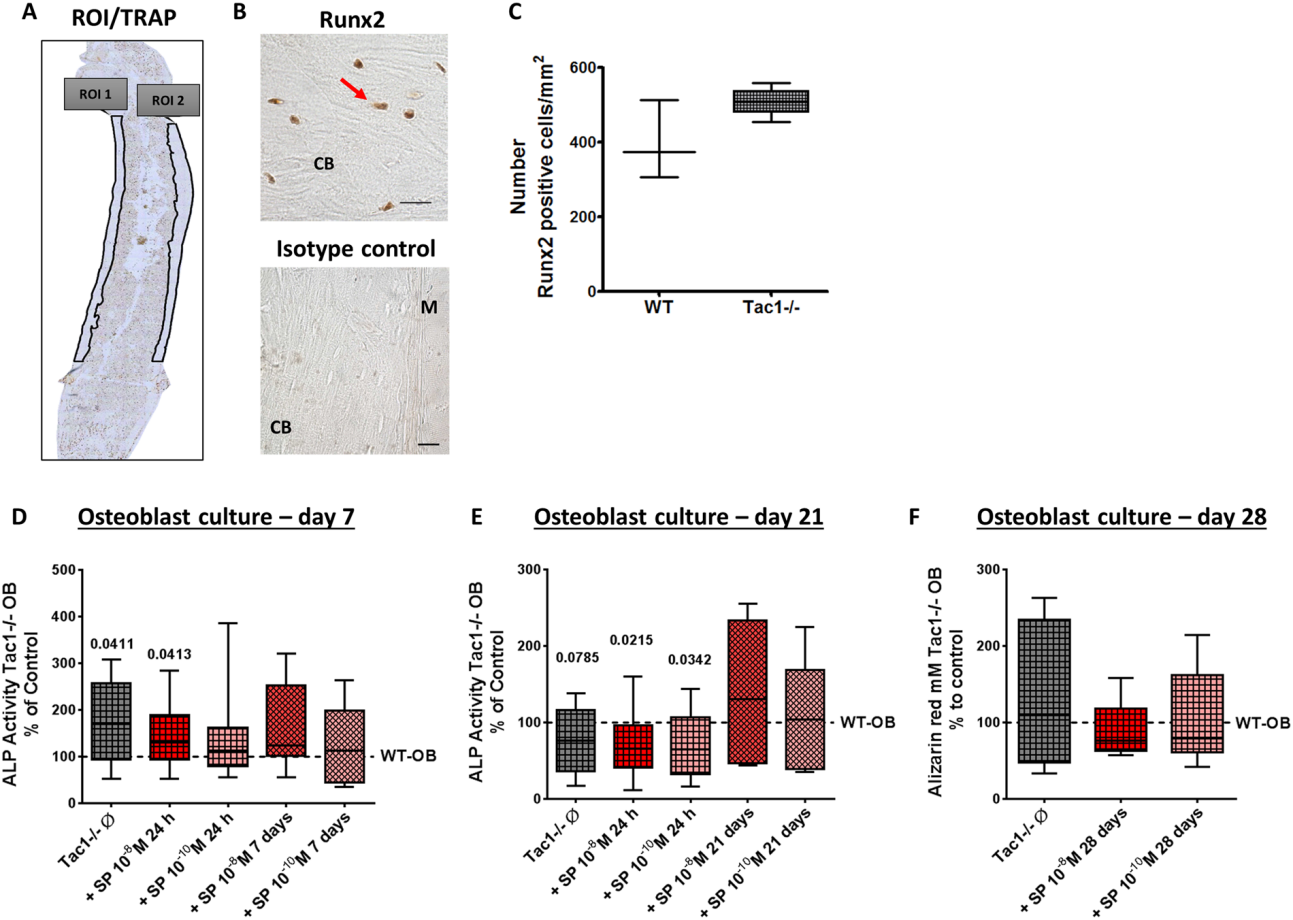
Immunohistological detection of Runx2-positive cells on paraffin sections of WT and Tac1−/− femora and *in vitro* activity of osteoblast cultures. (**A**) Representative image of the regions of interest (ROIs) set in Runx2 stained sections. Runx2-positive cells were counted only in cortical bone regions. (**B**) Representative images of Runx2-positive cells and isotype control in paraffin sections of WT femora. Scale bar = 20 µm. N = 3–4. M = Muscle; CB = Cortical bone. (**C**) Number of Runx2-positive cells/mm^2^ counted in ROIs of femoral paraffin sections of WT and Tac1−/− animals. N = 3–4. Comparison of osteoblast ALP activity (4 minute time point) after 7 (**D**) and 21 (**E**) days in osteogenic differentiation medium, w/o short-term (24 h) or long-term (7/21 days) stimulation of Tac1−/− osteoblasts with SP 10^−8^/10^−10^ M. Results of Tac1−/− animals were calibrated to WT controls (dotted line = 100% line). N = 8. Comparison of matrix mineralization (calcium deposition ability) by quantification of alizarin red staining of osteoblasts after 28 days in osteogenic differentiation medium, w/o long-term (28 days) stimulation of Tac1−/− osteoblasts with SP 10^−8^/10^−10^ M. Results of Tac1−/− animals were calibrated to WT controls (dotted line = 100% line). N = 6. Ø = no stimulation.

Number of Runx2-positive cells was counted on femoral paraffin sections to determine osteoblast number *in vivo* (representative images in Fig. 5A and B). Number of Runx2-positive cells in cortical bone area was similar in WT and Tac1−/− animals (Fig. 5C).

ALP enzyme activity was determined as a standard marker for bone formation. ALP activity was significantly higher in native Tac1−/− osteoblasts compared to WT during the very early differentiation phase (after 7 days culture in osteogenic medium). This was not affected by short-term (24 h) stimulation with 10^−8^ M SP. Short-term stimulation of Tac1−/− osteoblasts with 10^−10^ M SP as well as long-term stimulation with both concentrations of SP (10^−8^/10^−10^ M) resulted in ALP activity similar to WT level (Fig. 5D). After 21 days culture in osteogenic medium (late differentiation stage), ALP activity was by trend reduced in native Tac1−/− osteoblasts in comparison to WT. Short-term stimulation with SP (10^−8^/10^−10^ M) further diminished ALP enzyme activity in Tac1−/− osteoblasts. Long-term stimulation with both concentrations of SP (10^−8^/10^−10^ M) raised enzyme activity back to WT level (Fig. 5E).

Alizarin red staining as a marker for calcium deposition ability of mature osteoblasts was quantified after 28 days. The amount of alizarin red was comparable in native Tac1−/− osteoblast cultures compared to WT. Long-term stimulation with SP in both concentrations had no effect on calcium deposition ability of Tac1−/− osteoblasts (Fig. 5F).

### Expression of osteoclast-specific marker genes

**Figure 6:**
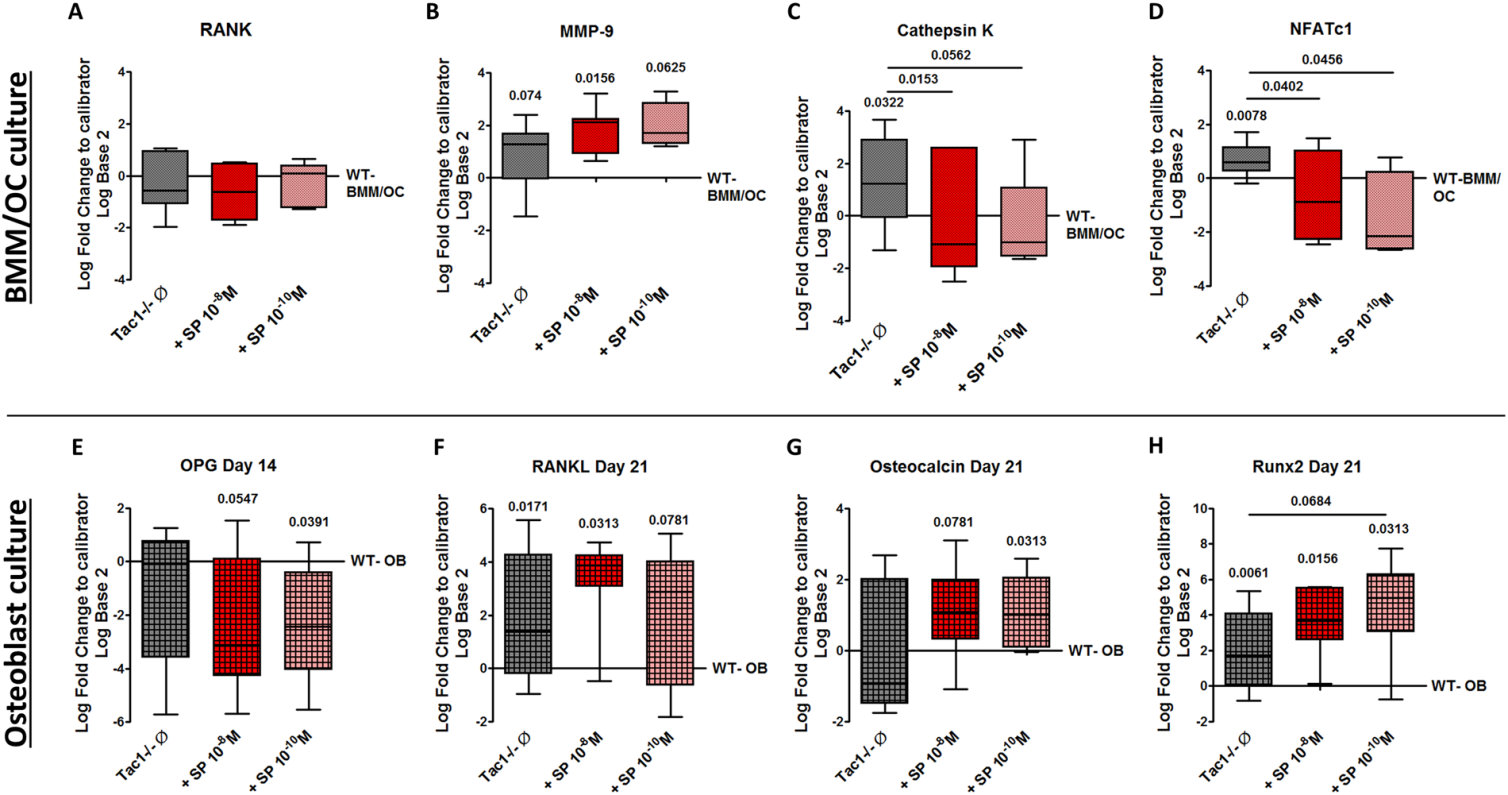
Osteoclast- and osteoblast-specific marker gene expression. Comparison of *RANK* (**A**), *MMP-9* (**B**), *CTSK* (cathepsin K; **C**) and *NFATC1* (**D**) gene expression in Tac1−/− to WT BMM/osteoclast cultures after 5 days of M-CSF and RANKL mediated differentiation. N = 5–11. Comparison of *TNFRSF11B* (OPG Day 14; **E**), *TNFSF11* (RANKL Day 21; **F**), *BGLAP* (osteocalcin day 21; **G**), *RUNX2* (Day 21; **H**) gene expression in Tac1−/− to WT osteoblasts after 14 (**E**) and 21 (**F**–**H**) days in osteogenic medium. N = 8–16. Results of RNA isolated from Tac1−/− cells were calibrated to RNA isolated from WT cells ( = x-axes/0-line). Ø = no stimulation.

The absence of SP resulted in changes of osteoclast-specific marker gene expression. Comparing gene expression of BMM/osteoclast cultures isolated from WT and Tac1−/− mice did not reveal differences in RANK gene expression (Fig. 6A). Gene expression of MMP 9 was upregulated by trend in native Tac1−/− BMM/osteoclast cultures and in cultures stimulated with 10^−10^ M SP but significantly upregulated after stimulation with 10^−8^ M SP (Fig. 6B). Cathepsin K and NFATc-1 gene expression were both upregulated in native Tac1−/− BMM/osteoclast cultures. Stimulation with 10^−8^ and 10^−10^ M SP reduced expression of both genes to WT level (Fig. 6C and D).

### Expression of osteoblast-specific marker genes

Loss of SP also affected gene expression of selected osteoblast-specific marker genes. Comparison of OPG gene expression revealed no differences between native Tac1−/− and WT osteoblasts at both culture time points. Stimulation of Tac1−/− osteoblasts with 10^−8^/10^−10^ M SP reduced OPG gene expression after 14 days culture (Fig. 6E) with no changes after 21 days (Supplementary file 2A). RANKL gene expression was not affected in Tac1−/− osteoblasts after 14 days (Supplementary file 2B) but was significantly higher in Tac1−/− osteoblasts, w/o 10^−8^ M SP after 21 days (Fig. 6F). Gene expression of osteocalcin was not affected in Tac1−/− osteoblasts w/o stimulation with SP (10^−8^/10^−10^ M) after 14 days culture (Supplementary file 2C). After 21 days culture, stimulation with 10^−8^ M SP increased osteocalcin gene expression by trend and significantly after stimulation with 10^−10^ M SP (Fig. 6G). Runx2 gene expression did not differ between WT osteoblasts and Tac1−/− osteoblasts w/o stimulation with 10^−8^/10^−10^ M SP after 14 days of osteogenic differentiation (Supplementary file 2D). After 21 days culture, Runx2 gene expression was upregulated in native Tac1−/− osteoblasts compared to WT osteoblast. Stimulation with both concentrations of SP (10^−8^/10^−10^ M) did not change Runx2 gene expression (Fig. 6H).

## Discussion

An increasing number of studies indicate that sensory nerve fibers modulate bone cell metabolism via neuropeptide-mediated signaling. The ability to react to SP-mediated signaling depends on the expression of the appropriate neurotransmitter receptors. Other groups reported earlier the presence of NK1R on different cell types of the musculoskeletal system, i.e osteoblasts, BMM and bone marrow derived stem cells^17,18,21^. Here, we demonstrated comparable protein expression of NK1R in BMM/osteoclasts and osteoblasts from SP-deficient Tachykinin 1 knockout and WT mice. Furthermore, for the first time we demonstrated that differentiated BMM/osteoclast cultures and osteoblasts isolated from WT mice endogenously produce and secrete SP at a picomolar range. This complements a previous study of our group which demonstrated SP production and secretion by murine costal chondrocytes kept in monolayer and pellet cultures^22^. Of note, our analysis revealed SP not only in serum of WT but also in serum of Tac1−/− animals at about 20–25% of WT values. We assume that we measured not SP but its relative hemokinin 1 (HK-1) which also belongs to the tachykinin family and is encoded by the tachykinin 4 gene^23^. HK-1 shows very high homology with SP on the amino acid level and was identified to be an agonist for NK1R binding with a pharmacological profile similar to SP^24^. As HK-1 is proposed to be expressed in the peripheral tissue -where SP is not expressed- as an endogenous peripheral SP-like endocrine/paracrine agonist, we have to take into account that the signal we measured in serum of Tac1−/− mice belongs to HK-1 and not to SP^25,26^. Nevertheless, these observations indicate that neuropeptide production not only of nerve fibers but also of resident bone and cartilage cells may contribute to control mechanisms during development in endochondral ossification and during adulthood in bone remodeling processes. Possibly, this might happen via an autocrine feedback loop as previously described in a model with human tenocytes which produced SP and modulated NK1R expression *in vitro* after mechanical loading^27^. Our previous observation that loss of SP impaired the process of fracture callus differentiation and bone remodeling in an adult model of endochondral ossification corroborates this hypothesis^20^.

Our data indicate that loss of SP seems to impair osteoblast survival *in vitro* during osteogenic differentiation. We detected a higher caspase 3/7 activity directly correlating to apoptosis rate in Tac1−/− osteoblasts during the early differentiation phase (after 14 days) without a rescue effect after short-term (24 h) stimulation with SP. In contrast, long-term stimulation normalized caspase 3/7 activity indicating that specifically persistent SP-mediated signaling has positive effects on osteoblast survival during early osteogenic differentiation. At a later time point (21 days) osteoblasts responded differently to SP which is in accordance to data from Yang *et al*. who reported that stimulation with 10^−10^ M SP prevented MC3T3 osteoblasts against serum deprivation-induced apoptosis^28^. Backman and colleagues also demonstrated positive effects of SP on cell survival^29^. In their studies, anti-Fas-induced decrease in cell viability of human tenocytes was prevented by simultaneous stimulation with SP. However, this effect was only observed when using high SP concentrations (10^−7^/10^−8^ M) which was comparable to our observation of short-term SP stimulation on osteoblast survival at late differentiation stages. We conclude a highly variable response of osteoblasts to SP dependent on stimulation period, culture time point and origin (SP-deficient versus WT (Suppl. Files 3A). Alterations in NK1R activation status, molecular structure or signaling pathways due to osteogenic differentiation stage and absence of its ligand, SP, may be the underlying molecular cause (see below).

In contrast, caspase 3/7 activity of BMM/osteoclast cultures was strongly reduced in the absence of SP however stimulation with SP increased caspase 3/7 activity. In this line, we demonstrated increased caspase 3/7 activity after stimulation of WT BMM/osteoclast cultures with high concentrations of SP, supporting our hypothesis that NK1R mediated SP-effects are cell type and concentration dependent (Suppl. Files 3D).

It has been reported that SP modulates proliferation in various cell types time- and dose-dependently. Proliferation of murine bone marrow stromal cells (BMSC) was enhanced by stimulation with 10^−8^ M SP during the first 3 days after seeding, but no differences were observed after 21 days when BMSC were differentiated to matrix building osteoblasts or to osteoclast precursor cells^18^. Long-term stimulation with SP induced chondrocyte proliferation in a pellet culture regimen until day 7 but decreased proliferation at later time points^22^. This is in line with our observations as long-term stimulation affected proliferation of SP-deficient osteoblasts in a similar manner - positively during early and negatively during late osteogenic differentiation. In contrast, loss of SP did not affect proliferation of BMM cultures or osteoblasts however, stimulation of SP-deficient BMM with SP clearly reduced proliferation. Notably, SP treatment of WT BMM and short-term stimulation of WT osteoblasts -which endogenously produce SP-did not affect proliferation of these cells (Suppl. Files 3B/E). These -together with the Caspase 3/7 activity data-indicate SP signaling via different intracellular pathways.

The NK1R belongs to the G protein-coupled receptor (GPCR) family and different intracellular signaling pathways have been described for NK1R and it’s ligand SP in various cell types, depending on the specific G proteins (G_αs_, G_αi_, G_α12/13_, G_α0_, G_αq_) bound to this receptor^30^. Possibly, lack of SP leads - due to changes in extracellular and intracellular conditions - to a compensation mechanism that results in a switch of G proteins, similarly to what has been described for the β_2_-adrenoceptor (β_2_AR). Baillie *et al*. demonstrated in a study with HEK293 cells and human cardiac myocytes that the G protein-coupling specificity of the β_2_AR can be reprogrammed in that it couples less well to G_αs_ and can then couple to G_αi_^31^. Another study from Jenei-Lanzl *et al*. demonstrated that G_αs_PCR signaling in human RA synovial cells switched to G_αi_PCR signaling under hypoxic conditions resulting in an unexpected proinflammatory signaling pathway^32^.

In addition, the human NK1R exists as a full-length and a C-terminally truncated form resulting in different signaling pathways and consequently different effects on metabolic cell functions^33–35^. Lai *et al*. demonstrated that full length NK1R mediates an increase in intracellular Ca^2+^ whereas a C-terminally truncated NK1R signals through Ca^2+^ independent mechanisms^34^. In our study, we did not detect significant changes of protein expression of the NK1R in the absence of SP. However, maybe murine bone cells also express the truncated receptor isoform besides the full-length receptor and loss of SP modulates expression of the truncated NK1R thus shifting signaling pathways from Ca^2+^ dependent to Ca^2+^ independent signal cascades or vice versa.

Differentiation to bone forming osteoblasts is characterized by an increase of ALP enzyme activity^36^ and osteocalcin production. ALP enzyme activity is induced in Tac1−/− osteoblasts at a very early differentiation time point (day 7) but was by trend reduced after 21 days and not rescued via short-term SP stimulation which was different for WT osteoblasts (Suppl. Files 3C). Only long-term SP stimulation was sufficient to replace the intracellular loss of SP. These differences in SP effects are reflected in literature. Adamus and Dabrowski reported reduced ALP enzyme activity already after 7 days when stimulating rat BMSC with SP^37^. In contrast, Wang *et al*. found a higher ALP enzyme activity in murine BMSC after 14 days of osteogenic differentiation when stimulating the cells with low concentrations of SP (10^−10^/10^−12^M)^18^. ALP enzyme activity is essential for bone matrix mineralization processes and early ALP activity seems to be a progression factor for osteoblast differentiation. This is in line with the data from Mei *et al*. who suggested that SP may enhance osteogenic differentiation of MC3T3-E1 cells^38^. Beck *et al*. demonstrated *in vitro* that osteoblast differentiation and matrix mineralization still occurred even when ALP activity was profoundly reduced but resulted in decreased mineral deposition. In contrast, we did not detect differences in calcium deposition ability of SP-deficient osteoblasts. Nevertheless, an impaired structural bone phenotype could possibly be a result of lower ALP activity of mature osteoblasts imperative for proper bone matrix formation^39,40^. Taken together, we assume that higher ALP enzyme activity at 7 days followed by higher apoptosis rate at 14 days of osteogenic differentiation possibly indicates an accelerated differentiation process accompanied by a reduction of viable metabolic active cells. The by trend reduced ALP enzyme activity after 21 days in mature Tac1−/− osteoblasts then adds to a reduction in bone formation processes and could explain partly the impaired biomechanical and structural properties of Tac1−/− femora^20^.

Another explanation for the impaired bone properties of Tac1−/− mice might be an increase in bone resorption activity of mature osteoclasts. However, we observed reduced cathepsin K enzyme activity in Tac1−/− BMM/osteoclast cultures even though gene expression of cathepsin K was increased. It was demonstrated that homozygote cathepsin (Cath) K−/− mice developed an osteopetrotic phenotype whereas the heterozygote animals appeared to be normal. Osteoclasts of Cath K−/− mice were still able to resorb the inorganic bone matrix but revealed defects in resorption and endocytosis of the organic parts^41^. These data suggest that reduced cathepsin K enzyme activity in Tac1−/− osteoclasts might be compensated. Possible mechanism could involve induction of osteoclastogenesis or activation of other proteolytic enzymes. Rifkin *et al*. demonstrated that the cysteine protease cathepsin L seems to be involved in bone resorption processes^42^. Kiviranta *et al*. measured an increase in MMP-9 and RANKL mRNA levels in bones of Cath K−/− mice^43^. These data corroborate our findings of increased MMP-9 mRNA levels in BMM/osteoclast cultures and increased RANKL mRNA levels in osteoblasts of Tac1−/− mice. In addition, NFATc1 mRNA expression, an important osteoclastic differentiation marker, was upregulated in Tac1−/− BMM/osteoclast cultures. Together, these observations point to induced osteoclastogenesis of Tac1−/− BMM possibly trying to compensate for reduced number of precursors in bone marrow.

Surprisingly, the number of TRAP-positive cells did not differ on femoral paraffin sections of Tac1−/− and WT mice whereas the fracture callus of Tac1−/− mice contained less osteoclasts as WT^20^. In our *in vitro* studies the number of TRAP-positive multinucleated osteoclasts was also reduced in Tac1−/− BMM/osteoclast cultures without being rescued via SP stimulation. As lower osteoclast numbers seem to be contradicting the structural changes in bone microarchitecture seen in the femora of Tac1−/− mice, we analyzed in addition osteoclast size. Indeed, the mean pixel size of Tac1−/− osteoclasts *in vitro* was higher indicating that loss of SP possibly increases the fusion potential of activated Tac1−/− BMM resulting in enlarged “super” osteoclasts able to resorb a larger bone area compared to WT conditions. In combination with the reduced apoptosis rate of Tac1−/− BMM/osteoclast cultures this could partially compensate the reduction in cathepsin K enzyme activity and might even increase bone resorption activity of individual osteoclasts. However, in combination with reduced macrophage and TRAP positive cell numbers plus reduced cathepsin K activity, the overall bone resorption capability might be still compromised in the absence of SP.

In our previous studies, where we compared BMM/osteoclast cultures of male SP-deficient mice with WT animals, apoptosis rate, cathepsin K activity and number of TRAP positive cells tend to be higher in the absence of SP. We suggest that the inconsistent results are due to the influence of sex hormones and possibly age. Sex steroids as estrogen have a profound effect on osteoblast and osteoclast metabolism^44^. Additionally, age-related changes in trabecular bone parameters seems to start at a young age (between 2–6 months) in C57Bl/6 J mice and the progression of this process seems to differ between female and male mice^45^. Together these data point to an important role of sex hormones as estrogens in bone turnover already at the age of 2 months.

An important observation of this study is that endogenously produced SP is critical for metabolic activities of bone cells ultimately determining bone turnover rate. Equally important is the observation that addition of exogenous SP not always rescued or influenced altered metabolic functions including proliferation, osteoclastogenesis and fusion, APL- and caspase activity. Moreover, the stimulation period played an important role. These observations point to a dose-, time- and cell type dependent effect of SP which is critical for intracellular signal transduction of the NK1R activating different signaling pathways eventually compromising bone cells ability to degrade and form bone matrix in a balanced manner.

Our data suggest that there exist neuropeptide mediated, cell autonomous changes in bone cell metabolism. Based on these findings, nerve fiber derived SP *in vivo* might provoke different effects on bone cell metabolism as the changes we observed *in vitro* in the absence of cellular derived SP. The different results of our short- and long-term stimulations with SP - which can also be depicted as external, nerve fiber derived SP – corroborate this hypothesis. Cellular and nerve fiber derived SP could possibly differ in relevance during bone development and bone remodeling as impaired bone microarchitecture in SP-deficient mice^20^ let suggest developmental changes when cellular derived SP is missing while an osteoporotic bone phenotype in adult rats after NK1R blockade^46^ indicate nerve fiber mediated effects on bone remodeling.

**Figure 7:**
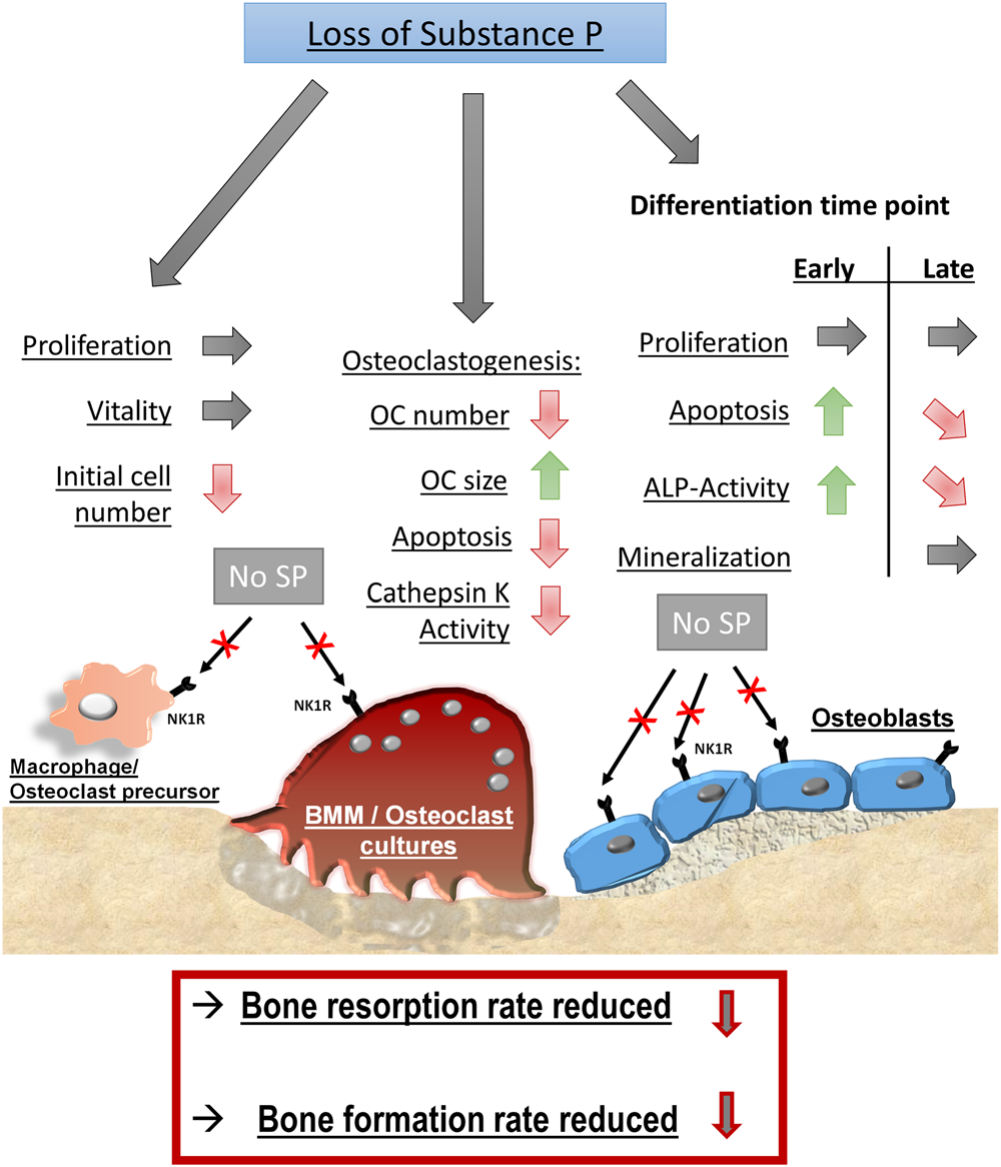
Proposed mechanism of how absence of SP affects metabolic activities of BMM/osteoclasts and osteoblasts. Macrophage/osteoclast precursor cultures: Loss of SP does not affect vitality and proliferation rate of macrophage cultures but results in reduced initial macrophage cell numbers under culture conditions. BMM/osteoclast cultures: Absence of SP does affect precursor fusion and differentiation to multinucleated osteoclasts resulting in less mature TRAP-positive osteoclasts with a larger size. Apoptosis rate is reduced in BMM/osteoclast cultures in the absence of SP but also bone resorption correlating to cathepsin K activity is negatively affected. Osteoblast cultures: Absence of SP mediated signaling has no effect on osteoblast proliferation during both differentiation time points. Loss of SP increases osteoblast apoptosis rate during early differentiation (14 days) time point whereas apoptosis rate was reduced by trend during late differentiation time point (21 days). ALP-activity was increased during very early differentiation time point (7 days) whereas ALP-activity in SP-deficient osteoblast cultures was reduced by trend at the late differentiation stage (21 days). Matrix mineralization (calcium deposition ability) of mature osteoblasts (28 days) was not affected in the absence of SP. Conclusion: Loss of SP mediated signaling presumably results in a slightly reduced bone resorption rate but also in a slightly reduced bone formation rate. As bone resorption is faster than bone formation, we suggest a net bone loss in the absence of SP.

In summary, our data indicate that in the absence of SP bone resorption rate might be slightly reduced. Concomitantly, we suggest that bone formation rate is more critically reduced, due to faster osteoblast precursor differentiation accompanied by a reduction of viable metabolic active cells during the early differentiation phase. A reduced bone mineralization rate in the late differentiation phase adds to an impaired bone formation rate (Fig. 7). Taken together, as the bone resorption process is much faster than the bone formation process, these findings result in a net bone loss which likely explains the impaired biomechanical and structural bone parameters that has been described in Tac1−/− mice^20^.

## Material and Methods

### Animals

To characterize the effects of SP loss on BMM and osteoblasts metabolism, female Tachykinin 1 (Tac1)-deficient mice (age: 9–12 weeks; 18 mice were used for cell isolation; 4 mice were used for analysis of paraffin embedded bone sections) that harbor a targeted mutation in the Tachykinin 1 gene on a C57Bl/6 J background^47^ were used for bone cell isolation. Female age-matched wild type (WT) C57Bl/6 J mice (Charles River, Sulzfeld, Germany; 25 mice were used for cell isolation; 3 mice were used for analysis of paraffin embedded bone sections) served as control group. Animals were kept under standardized conditions with free access to food and water. All animals were used for organ extraction in agreement with the local veterinary administration (City Regensburg, Umweltamt, Dept. Veterinärwesen und Verbraucherschutz) and in accordance with the ethical committee appointed by the local authority (Regierung von Unterfranken, Bayern) controlling animal experimental usage (Az.: 54-2532.1-23/14). All experimental protocols/methods were approved and carried out in accordance with the relevant guidelines and regulations of the above named committees.

### Isolation of primary murine bone marrow macrophages and osteoblasts

BMM and osteoblast-like cells were prepared according to established protocols with minor modifications^20,48,49^. Cells from different animals were never pooled. In brief, for preparing macrophage cultures, long bones were removed, cleaned from surrounding tissue and washed with PBS. Epiphyses were cut off and bone marrow was flushed out with medium (αMEM, #M4526, Sigma-Aldrich, Taufkirchen, Germany). Bone marrow cells were pelleted by centrifugation. Erythrocytes were lysed by hypotonic shock, remaining cells were centrifuged and resuspended in medium supplemented with 10% FCS, 1% Pen/Strep, 2% GlutaMAXTM-I (100×, #35050-38, Gibco Life Technologies, Darmstadt, Germany), 20 ng/ml M-CSF (#315-02, PeproTech, Hamburg, Germany) and cultured in petri dishes for 2 days. For preparing osteoblast cultures, diaphyses of long bones were cut into bone chips (2 × 2 mm) and cleaned with collagenase solution (175 U/ml Collagenase Type II in PBS, Worthington Biochemical Corp., Lakewood, USA) twice for 20 min at 37 °C. Bone chips were washed with growth medium (DMEM low glucose, #31885–023, Gibco Life Technologies, Darmstadt, Germany) and cultured in T 25 culture flasks as explant culture (=E1) in growth medium supplemented with 10% FCS, 1% Pen/Strep, 100 µM ascorbic acid 2-phosphate (Sigma, Saint Louis, USA). Osteoblast-like cells started to migrate out of bone chips (cells in passage 0 of explant culture E1 = E1P0) after 5 days and were cultured until confluency (14 ± 2 days).

### Cell differentiation assays

To generate mature osteoclasts, BMM were seeded and cultured for 5 days in differentiation medium (α-MEM + 10% FCS, 1% Pen/Strep, 2% GlutaMax, 20 ng/ml M-CSF and 10 ng/ml RANKL (#462-TEC/CF, R&D Systems)). After 5 days, differentiation capacity was analyzed by staining of BMM/osteoclast cultures, seeded in triplicates in a 96 well plate, for tartrate resistant acid phosphatase (TRAP), a standard marker enzyme for osteoclasts, with the Acid Phosphatase, Leukocyte Kit (#387 A, Sigma-Aldrich, St. Louis, USA). Differentiation capacity was determined by counting TRAP positive osteoclasts with ≥3 nuclei. To induce terminal osteoblast differentiation, osteoblast-like cells (E1P0) were harvested, seeded for the experiments and cultured (passage 1 after explant culture = E1P1) in osteogenic medium (MEM, #M8042, Sigma-Aldrich, Taufkirchen, Germany) supplemented with 10% FCS, 1% Pen/Strep, 4 mM GlutaMax, 100 µM ascorbic acid 2-phosphate, 10 mM β-glycerophosphate (#G9422, Sigma-Aldrich, Steinheim, Germany), 100 nM dexamethasone (#D2915, Sigma-Aldrich, Steinheim, Germany) for 7, 14, 21 or 28 days depending on assay conditions.

### Determination of cell numbers and outgrowth rate

After 2 days, petri dishes with adhered bone marrow cells were washed twice with ice cold PBS and suspension cells were sucked off. Remaining adhered BMM were detached with 0.02% EDTA in PBS (4 °C), centrifuged and cell pellets were resuspended in medium. Cell number was determined using a Cedex XS Analyzer (Roche Diagnostics).

To compare outgrowth rates of osteoblast-like cells from bone, diaphysis of femora and tibia were cut into bone chips, weighed and cultured in 6-well plates for 5, 8 or 11 days. Bone chips were removed, number of outgrown cells was analyzed using crystal violet staining procedure. Briefly, cells were washed with PBS, fixated with 1% glutaraldehyde and stained with 0.02% crystal violet. Cells were then washed with water and incubated with 70% ethanol for 3 h on a plate shaker to solubilize the dye. An aliquot was measured at 595 nm with an ELISA plate reader. Results were calculated as OD (crystal violet, 595 nm)/mg bone.

### Endogenous SP production in bone cells

Bone cells isolated from WT animals were kept for 24 h under serum-free conditions (osteoclasts from day 4 to 5; osteoblasts from days 13 to 14 and from 20 to 21). Some cultures were incubated with IL-1β (0.5 ng/ml) to mimic inflammatory conditions. Serum was generated from blood samples obtained from WT and Tac1−/− animals. SP concentration in serum and cell culture supernatants of osteoclasts and osteoblasts was analyzed with a substance P ELISA kit (Enzo Life Sciences (ELS) AG, Lausen, Switzerland; Detection Limit 9.76 pg/ml) according to manufacturer’s instructions.

### Immunofluorescence staining

Osteoclasts and osteoblasts prepared from WT and Tac1−/− animals were cultured in chamber slides (Falcon #354108, Corning Incorporated, NY, USA) as described. Osteoclasts were stained for NK1R expression after 5 days of osteoclastic differentiation, osteoblasts after 21 days of osteogenic differentiation. Cells were washed with PBS and fixed with acetone (−20 °C) for 10 min at 4 °C followed by two washing steps with PBS, each for 5 min at RT. Unspecific binding sites were then blocked with 1% BSA and 5% normal goat serum (NGS) in PBS for 60 min at 37 °C. For immunofluorescence staining, cells were incubated with a primary antibody against NK1R (ab183713; 1.8 µg/ml, Abcam, Cambridge, UK) in blocking solution over night at 4 °C. Secondary antibodies conjugated to Alexa568 (#A10042, Invitrogen, Karlsruhe, Germany) were used for primary antibody detection on osteoclasts and secondary antibodies conjugated to Alexa 488 (#4412, Cell Signaling, New England Biolabs GmbH, Frankfurt a.M., Germany) were used for osteoblasts. DAPI (Invitrogen) was applied for nuclei detection and slides were embedded in Fluorescent Mounting Media (Dako North America Inc., Carpinteria, CA, USA). Pictures were taken with an Olympus BX61 microscope (Olympus Deutschland GmbH, Hamburg, Germany) with 40-fold magnification.

### Western Blot analysis

Cells in 6-Well plates were washed with PBS and lysed with RIPA buffer (Thermo Scientific, Rockford, IL, USA) containing proteinase inhibitors (Complete Mini, Roche Diagnostics, Mannheim, Germany). Total protein content in cell lysates was quantified by the BCA method (Thermo Scientific, Rockford, IL, USA). Aliquots of 10 µg of total protein were mixed with SDS-sample buffer (+DTT), heated for 5 min at 95 °C and loaded onto a 12% SDS-PA gel. After separation for 130 min (120 V), proteins were blotted to nitrocellulose membranes (AmershamTM ProtranTM 0.45 µm, GE Healthcare Europe GmbH, Freiburg, Germany) for 90 min (120 mA). Membranes were blocked with 5% dry milk in T-TBS buffer for 1 h at RT followed by incubation with primary antibody against NK1R over night at 4 °C (ab183713, 1:20.000, Abcam, Cambridge, UK). β-actin (ab8227, 1:5000, Abcam) was used as loading control. Subsequently, membranes were washed with PBS and incubated with a horseradish peroxidase-coupled secondary antibody (#711−036–152, 1:10.000, Jackson Immuno Research, West Grove, PA, USA). ECL detection reagent (Thermo scientific) was used for protein detection and Photoshop CS4 was used for color inversion and measurement of band pixel size and median grey value. Pixel size of each band (NK1R and β-actin) was multiplied with the associated median grey value. To calculate a correction factor, the values from one β-actin band of one WT-control sample on each blot was set as 1 (=β-actin correction value). The correction factor of the remaining WT and Tac1−/− samples on each blot were calculated as followed: ((100/“β-actin correction value”) *β-actin WT or Tac1−/− sample). Next, NK1R expression was normalized to β-actin by multiplication with the respective correction factor. Finally, results of Tac1−/− animals were calculated to controls (=100%).

### Stimulation of bone cells with SP

For all experiments, BMM/osteoclasts and osteoblast-like cell cultures of Tac1−/− and also partly WT (Suppl. Files 2) mice were stimulated with 10^−8^ and 10^−10^ M of recombinant SP (#S6883, Sigma-Aldrich Chemie GmbH, Schnelldorf, Germany), either for the last 24 h of culture time or for entire culture period with every medium exchange (only Tac1−/− cultures) to mimic neurotransmitter milieu predominant in WT tissues.

### Cell spreading assay

BMM or osteoblast-like cells were seeded in 96-well plates (5000/well) and cultured in growth medium for 4 h. After washing with PBS, number of adherent cells was quantitated using crystal violet staining procedure as described before (see outgrowth rate).

### Cell proliferation assay

BMM were cultured for 24 h in a 96-well plate (5000/well). Cells were synchronized by 24 h serum deprivation, afterwards cell growth was initiated over night by adding serum containing growth medium. Proliferation was quantified by determining BrdU incorporation after 24 h (Cell Proliferation ELISA, BrdU (colorimetric), Roche, Diagnostics GmbH, Mannheim, Germany).

Osteoblast-like cells were seeded in a 96-well plate (5000/well) and cultured in osteogenic medium for either 13 or 20 days. Subsequently, proliferation was determined via BrdU incorporation for 24 h (Cell Proliferation ELISA, Roche).

### Caspase 3/7-activity assay

To analyze apoptotic effects, we determined caspase 3/7-activity using Caspase-Glo® 3/7 Assay (Promega GmbH, Mannheim, Germany) according to manufacturer’s protocol. Caspase 3/7-activity was quantified in BMM/osteoclast cultures after 5 days in differentiation medium and in osteoblast cultures after 14 and 21 days in osteogenic differentiation medium.

### Immunohistochemistry and morphometry

To determine osteoclast and osteoblast numbers from long bones of WT control and Tac1−/− mice (age: 12 weeks) *in vivo*, left femora of WT- and Tac1−/− animals were dissected, fixed with paraformaldehyde in PBS for 24 h and decalcified in 20% ethylene diaminetetraacetic acid (EDTA, pH 7.3; Roth, Karlsruhe, Germany) for 4–6 weeks. Femora were dehydrated, embedded in paraffin and sections of 5 µm thickness were prepared for immunohistochemical staining. To analyze osteoclast numbers, three sagittal paraffin sections per mouse femur were dewaxed, rehydrated and osteoclasts were visualized by TRAP staining (#387 A, Sigma-Aldrich). Overview images were photographed using the TissueFaxSi plus system (10-fold magnification, TissueGnostics: INST89/341–1FUGG, Vienna, Austria) and total bone area was determined (Fig. 4A, HistoQuest Software, TissueGnostics). All TRAP positive cells were counted using 20-fold magnification and subsequently osteoclast numbers/mm^2^ were calculated.

To determine osteoblast numbers in the cortical bone area, three sagittal paraffin sections per mouse femur were dewaxed, rehydrated and endogenous peroxidase was blocked by incubation for 5 min in 3% hydrogen peroxide (Roth). Sections were washed twice with TBS-T for 5 min and antigen retrieval was conducted at 60 °C in a water bath for 24 h in sodium citrate buffer (10 mM sodium citrate, 0.05% Tween 20, pH 6.0). Sections were incubated with 1% BSA and 5% swine serum for 1 h at RT to block unspecific binding sites and subsequently incubated with primary antibodies against Runx2 (1:1000, ab192256, Abcam) over night at 4 °C in blocking solution and afterwards washed with PBS. Secondary biotinylated antibody (#E0431, DAKO, 1:500, 37 °C, 1 hour) was used for detection followed by incubation with Strepavidin-HRP (#P0397, DAKO, 1:300, 37 °C, 1 h). Afterwards sections were washed twice with PBS and incubated with DAB Enhanced Liquid Substrate System (# D3939, Sigma-Aldrich, Steinheim, Germany) at RT for 10 min. Finally sections were embedded in Dako Glycergel Mounting Medium (#C0563, Dako). Overview images were photographed (20-fold magnification, TissueFaxSi plus, TissueGnostics) and cortical bone area was determined (Fig. 4B) using HistoQuest software (TissueGnostics). Stained cells in cortical bone area were counted and results were calculated as Runx2 positive cells/mm^2^.

### Bone resorption, bone formation activity and matrix mineralization assay

As a marker for bone resorption activity of mature osteoclasts cathepsin K enzyme activity was analyzed in BMM/osteoclast cultures as described before^20,50^.

Bone formation activity was analyzed by quantifying intracellular alkaline phosphatase (ALP) activity using the QuantiChromTM Alkaline Phosphatase Assay Kit (DALP-250, BioAssay Systems, Hayward, CA, USA) according to manufacturer’s instructions.

Matrix mineralization (Calcium deposition) of osteoblasts was analyzed by quantification of alizarin red staining. Briefly, after 28 days cells were washed with PBS and fixed with 2.5% glutaraldehyde (in PBS) for 10 min at RT. Afterwards cells were washed 2x with PBS (pH 4.2) and calcium deposition was stained with 2% alizarin red solution for 20 min at 37 °C. After 2 washing steps (PBS) cell layer was incubated with 10% acetic acid for 30 min, gently scraped and total well content was transferred to a fresh tube. After heating to 85 °C for 10 min, samples were incubated on ice for 5 min, centrifuged at 20,000 × g for 15 min and pH was adjusted using 10% ammonium hydroxide (4.1–4.5). OD of samples and standard curve was measured at 405 nm and amount of alizarin red was calculated (mM alizarin red).

### Gene expression

Absolutely RNA Nanoprep Kit (Agilent Technologies, Waldbronn, Germany) was used for cell lysis and RNA extraction of osteoblasts according to manufacturer’s protocol (70% ethanol was used instead of 80% sulfolane). For Osteoclasts Absolutely RNA Miniprep Kit (Agilent Technologies, Waldbronn, Germany) was used. Single stranded cDNA was transcribed with the AffinityScript cDNA Synthesis Kit (Agilent Technologies, Waldbronn, Germany). For quantitative Real-time PCR, 20 ng cDNA per reaction mix was used with Brilliant II SYBR® Green QPCR Master Mix (Agilent Technologies, Waldbronn Germany). Relative gene expression was normalized to GAPDH and calibrated to WT control RNA (Log fold change to calibrator, Log base 2). Primers used are listed in Table 1.

**Table 1:** Primers used for gene expression analysis.

PRIMER	FORWARD: 5′-3′ SEQUENZ REVERSE: 5′-3′ SEQUENZ	AMPLICON SIZE [BP]
TNFRSF11A (RANK)	5′-GCTCCTGAAATGTGGACCAT-3′	241
	5′-CACGATGATGTCACCCTTGA-3′	
MMP9	5′- GAAGGCAAACCCTGTGTGTT-3′	228
	5′-AGAGTACTGCTTGCCCAGGA-3′	
CTSK (CATHEPSIN K)	5′- AGGCGGCTATATGACCACTG-3′	176
	5′-TCTTCAGGGCTTTCTCGTTC-3′	
NFATC1	5′-GGTGCTGTCTGGCCATAACT-3′	232
	5′-GAAACGCTGGTACTGGCTTC-3′	
TNFRSF11B (OPG)	5′-CGAGTGTGTGAGTGTGAGGAA-3′	112
	5′-TGTTTCGCTCTGGGGTTC-3′	
TNFSF11 (RANKL)	5′-TGGAAGGCTCATGGTTGGAT-3′	225
	5′-AGCAAATGTTGGCGTACAGG-3′	
BGLAP (OSTEOCALCIN)	5′-TGAGGACCATCTTTCTGCTCA-3′	108
	5′-TGGACATGAAGGCTTTGTCA-3′	
RUNX2	5′-CCTCTGACTTCTGCCTCTGG-3′	105
	5′-ATGAAATGCTTGGGAACTGC-3′	
GAPDH (HOUSE KEEPER)	5′-AACTTTGGCATTGTGGAAGG-3′	223
	5′-ACACATTGGGGGTAGGAACA-3′	

Primers were design with ncbi, Primer 3 Software (Sourceforge.net) and Ensembl (genome browser) and purchased from Microsynth AG (Balgach, Switzerland).

### Statistical Methods

Graph Pad Prism 6.0 software was used for statistical analysis and Graph preparation. All data are represented as median ±5/95 percentile. Wilcoxon signed-rank test was applied when WT controls were set to 100%. Differences in median were analyzed using two-tailed Mann-Whitney U-test. P values less than 0.05 were considered as significant.

### Data availability statement

The datasets generated and analyzed during the current study are available from the corresponding author on reasonable request.

## Electronic supplementary material

Supplementary files

## References

[1] Raggatt LJ, Partridge NC (2010). Cellular and Molecular Mechanisms of Bone Remodeling. Journal of Biological Chemistry.

[2] Hadjidakis DJ, Androulakis II (2006). Bone Remodeling. Annals of the New York Academy of Sciences.

[3] Raisz LG (1999). Physiology and Pathophysiology of Bone Remodeling. Clinical Chemistry.

[4] Sims NA, Gooi JH (2008). Bone remodeling: Multiple cellular interactions required for coupling of bone formation and resorption. Seminars in cell & developmental biology.

[5] Rubin, J. & Greenfield, E. In *Bone Resorption* Vol. 2 *Topics in Bone Biology* (eds Felix Bronner, MaryC Farach-Carson, & Janet Rubin) Ch. 1, 1–23 (Springer London, 2005).

[6] Teitelbaum SL (2000). Bone Resorption by Osteoclasts. Science.

[7] Takami M, Woo JT, Nagai K (1999). Osteoblastic cells induce fusion and activation of osteoclasts through a mechanism independent of macrophage-colony-stimulating factor production. Cell and tissue research.

[8] Hsu H (1999). Tumor necrosis factor receptor family member RANK mediates osteoclast differentiation and activation induced by osteoprotegerin ligand. Proc.Natl.Acad.Sci.USA.

[9] Eriksen EF (2010). Cellular mechanisms of bone remodeling. Rev Endocr Metab Disord.

[10] Pittenger MF (1999). Multilineage Potential of Adult Human Mesenchymal Stem Cells. Science.

[11] Komori T (1997). Targeted disruption of Cbfa1 results in a complete lack of bone formation owing to maturational arrest of osteoblasts. Cell.

[12] Robling AG, Castillo AB, Turner CH (2006). Biomechanical And Molecular Regulation Of Bone Remodeling. Annual Review of Biomedical Engineering.

[13] Murshed M, Harmey D, Millán JL, McKee MD, Karsenty G (2005). Unique coexpression in osteoblasts of broadly expressed genes accounts for the spatial restriction of ECM mineralization to bone. Genes & Development.

[14] Grassel S (2014). The role of peripheral nerve fibers and their neurotransmitters in cartilage and bone physiology and pathophysiology. Arthritis Research & Therapy.

[15] Bjurholm A, Kreicbergs A, Brodin E, Schultzberg M (1988). Substance P- and CGRP-immunoreactive nerves in bone. Peptides.

[16] Hukkanen M (1992). Distribution of nerve endings and sensory neuropeptides in rat synovium, meniscus and bone. Int J Tissue React.

[17] Goto T (2007). Substance P stimulates late-stage rat osteoblastic bone formation through neurokinin-1 receptors. Neuropeptides.

[18] Wang L (2009). *Zhao,R*.,Shi,X.,Wei,T., Halloran,B.P., Clark,D.J., Jacobs,C.R. & Kingery,W.S.Substance P stimulates bone marrow stromal cell osteogenic activity, osteoclast differentiation, and resorption activity *in vitro*. Bone.

[19] Mori T (1999). Substance P regulates the function of rabbit cultured osteoclast; increase of intracellular free calcium concentration and enhancement of bone resorption. Biochemical and biophysical research communications.

[20] Niedermair T (2014). Absence of substance P and the sympathetic nervous system impact on bone structure and chondrocyte differentiation in an adult model of endochondral ossification. Matrix Biology.

[21] Goto T, Yamaza T, Kido MA, Tanaka T (1998). Light- and electron-microscopic study of the distribution of axons containing substance P and the localization of neurokinin-1 receptor in bone. Cell and tissue research.

[22] Opolka A, Straub R, Pasoldt A, Grifka J, Grassel S (2012). Substance P and norepinephrine modulate murine chondrocyte proliferation and apoptosis. Arthritis and rheumatism.

[23] Zhang Y, Lu L, Furlonger C, Wu GE, Paige CJ (2000). Hemokinin is a hematopoietic-specific tachykinin that regulates B lymphopoiesis. Nature immunology.

[24] Morteau O, Lu B, Gerard C, Gerard NP (2001). Hemokinin 1 is a full agonist at the substance P receptor. Nat Immunol.

[25] Bellucci F (2002). Pharmacological profile of the novel mammalian tachykinin, hemokinin 1. British journal of pharmacology.

[26] Page NM (2004). Hemokinins and endokinins. Cell Mol Life Sci.

[27] Backman LJ, Fong G, Andersson G, Scott A, Danielson P (2011). Substance P is a mechanoresponsive, autocrine regulator of human tenocyte proliferation. PloS one.

[28] Yang J (2017). The fast track to canonical Wnt signaling in MC3T3-E1 cells protected by substance P against serum deprivation-induced apoptosis. Cell Biology International.

[29] Backman LJ, Danielson P (2013). Akt-mediated anti-apoptotic effects of substance P in Anti-Fas-induced apoptosis of human tenocytes. Journal of cellular and molecular medicine.

[30] Garcia-Recio S, Gascon P (2015). Biological and Pharmacological Aspects of the NK1-Receptor. BioMed research international.

[31] Baillie GS (2003). D. beta-Arrestin-mediated PDE4 cAMP phosphodiesterase recruitment regulates beta-adrenoceptor switching from Gs to Gi. Proceedings of the National Academy of Sciences of the United States of America.

[32] Jenei-Lanzl Z, Zwingenberg J, Lowin T, Anders S, Straub RH (2015). Proinflammatory receptor switch from Gαs to Gαi signaling by β-arrestin-mediated PDE4 recruitment in mixed RA synovial cells. Brain, Behavior, and Immunity.

[33] Steinhoff, M. S., von Mentzer, B., Geppetti, P., Pothoulakis, C. & Bunnett, N. W. *Tachykinins and Their Receptors: Contributions to Physiological Control and the Mechanisms of Disease*. Vol. 94 (2014). 10.1152/physrev.00031.2013 PMC3929113 24382888

[34] Lai JP (2008). Differences in the length of the carboxyl terminus mediate functional properties of neurokinin-1 receptor. Proceedings of the National Academy of Sciences of the United States of America.

[35] Munoz, M., Munoz, M. F. & Ayala, A. Immunolocalization of Substance P and NK-1 Receptor in ADIPOSE Stem Cells. *Journal of cellular biochemistry*, 10.1002/jcb.26134 (2017). 10.1002/jcb.26134 28500728

[36] Aubin JE, Liu F, Malaval L, Gupta AK (1995). Osteoblast and chondroblast differentiation. Bone.

[37] Adamus MA, Dabrowski ZJ (2001). Effect of the neuropeptide substance P on the rat bone marrow-derived osteogenic cells *in vitro*. Journal of cellular biochemistry.

[38] Mei G (2014). Substance P Activates the Wnt signal transduction pathway and enhances the differentiation of mouse preosteoblastic MC3T3-E1 cells. Int J Mol Sci.

[39] Beck GR, Sullivan EC, Moran E, Zerler B (1998). Relationship between alkaline phosphatase levels, osteopontin expression, and mineralization in differentiating MC3T3-E1 osteoblasts. Journal of cellular biochemistry.

[40] Torii Y, Hitomi K, Yamagishi Y, Tsukagoshi N (1996). Demonstration Of Alkaline Phosphatase Participation In The Mineralization Of Osteoblasts By Antisense Rna Approach. Cell Biology International.

[41] Gowen M (1999). Cathepsin K Knockout Mice Develop Osteopetrosis Due to a Deficit in Matrix Degradation but Not Demineralization. Journal of Bone and Mineral Research.

[42] Rifkin BR (1991). Cathepsin B and L activities in isolated osteoclasts. Biochemical and biophysical research communications.

[43] Kiviranta R (2005). Impaired bone resorption in cathepsin K-deficient mice is partially compensated for by enhanced osteoclastogenesis and increased expression of other proteases via an increased RANKL/OPG ratio. Bone.

[44] Khosla S, Monroe DG (2018). Regulation of Bone Metabolism by SexSteroids. Cold Spring Harbor perspectives in medicine.

[45] Glatt V, Canalis E, Stadmeyer L, Bouxsein ML (2007). Age-related changes in trabecular architecture differ in female and male C57BL/6J mice. Journal of bone and mineral research: the official journal of the American Society for Bone and Mineral Research.

[46] Kingery W (2003). A substance P receptor (NK1) antagonist enhances the widespread osteoporotic effects of sciatic nerve section. Bone.

[47] Zimmer A (1998). Hypoalgesia in mice with a targeted deletion of the tachykinin 1 gene. Proc. Natl. Acad. Sci. USA.

[48] Dillon JP (2012). Primary human osteoblast cultures. Methods in molecular biology.

[49] Orriss IR, Arnett TR (2012). Rodent osteoclast cultures. Methods in molecular biology.

[50] Wittrant Y (2003). Regulation of osteoclast protease expression by RANKL. Biochem. Biophys. Res. Commun..

