# Supplementary material for: Substance P modulates bone remodeling properties of murine osteoblasts and osteoclasts
Source: Sci Rep. 2018 Jun 15;8:9199. doi: 10.1038/s41598-018-27432-y (PMC6003941; doi:10.1038/s41598-018-27432-y)
Supplement: Supplementary file 1 — Supplementary files [file 41598_2018_27432_MOESM1_ESM.docx]

**Substance P modulates bone remodeling properties of murine osteoblasts and osteoclasts**

Tanja Niedermair^1,2^, Stephan Schirner^2^, Raphael Seebröker^2^, Rainer H. Straub^3^, Susanne Grässel^1,2^

^1^ Department of Orthopaedic Surgery, University of Regensburg, Germany

^2^ Department of Orthopaedic Surgery, Experimental Orthopaedics, Centre for Medical Biotechnology, University of Regensburg, Germany

^3^ Experimental Rheumatology and Neuroendocrine-Immunology; Department of Internal Medicine; University of Regensburg, Germany

*Corresponding author:

Susanne Grässel, Ph.D.

Dept. of Orthopaedic Surgery

University of Regensburg

ZMB / BioPark 1

Am BioPark 9

93053 Regensburg

Germany

Tel: ++49 941-943-5065

Fax: ++49-941-943-5066

Email: [susanne.graessel@ukr.de](mailto:susanne.graessel@ukr.de)

Supplementary Information


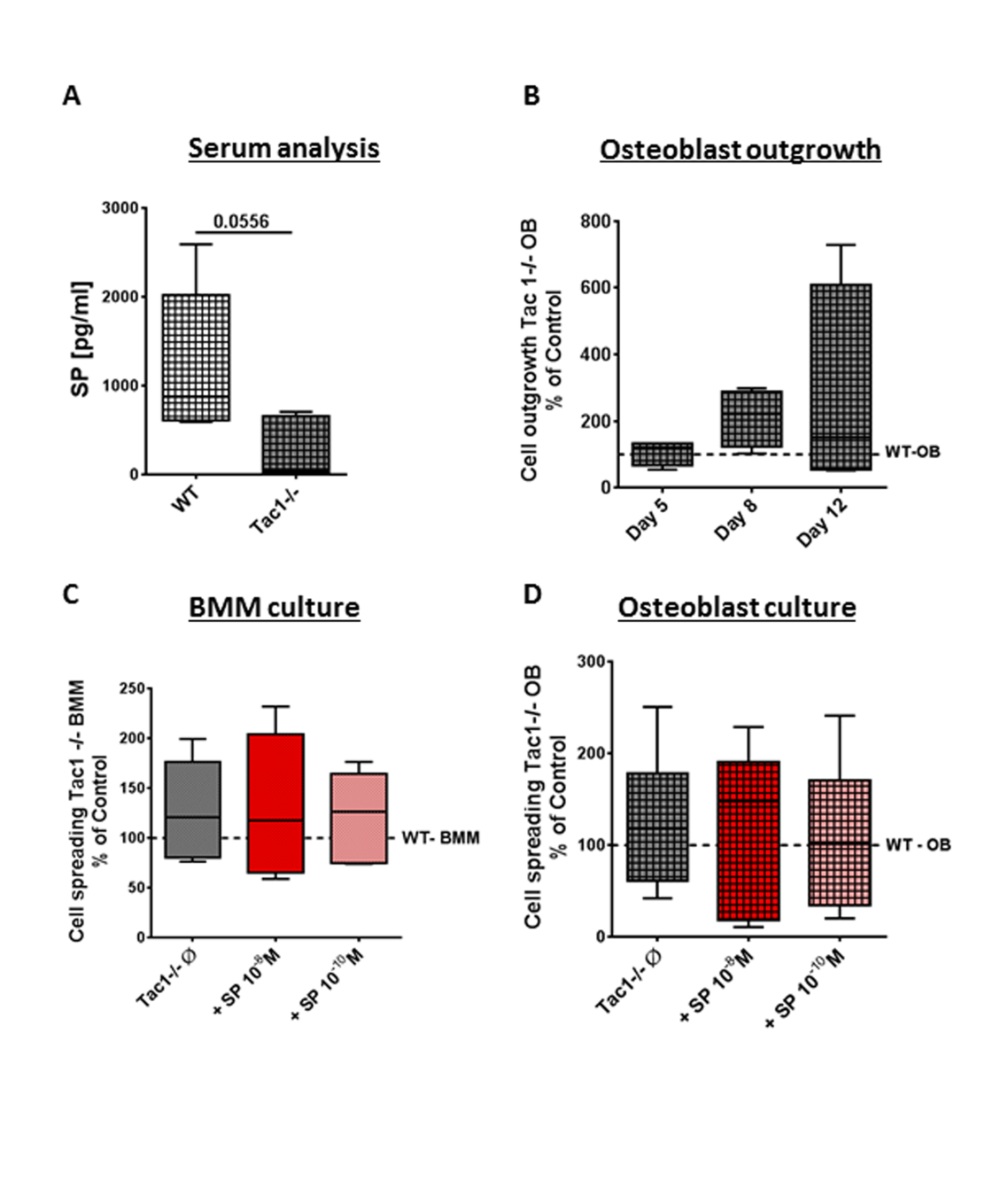


**Supplementary file 1: Serum neuropeptide concentration, cell outgrowth of osteoblasts and cell spreading of BMM/osteoclast cultures and osteoblasts**

(A) ELISA analysis of SP neuropeptide concentration [pg/ml] in serum samples of WT and Tac1-/- animals. N=5. (B)Osteoblast-like cell outgrowth from bone explants of WT and Tac1-/- mice, cultured in growth medium for 5, 8 and 12 days. Crystal violet staining intensity was measured and normalized to explant weight [g]. N=4.

Cell spreading of BMM (C) and osteoblasts (D) isolated from WT and Tac1-/- bone marrow and bone explants after 4 hours, w/o stimulation of Tac1-/- cultures with SP 10-8/10-10 M.

Results of Tac1-/- animals were calibrated to WT controls (dotted line = 100% line). N=8-12.


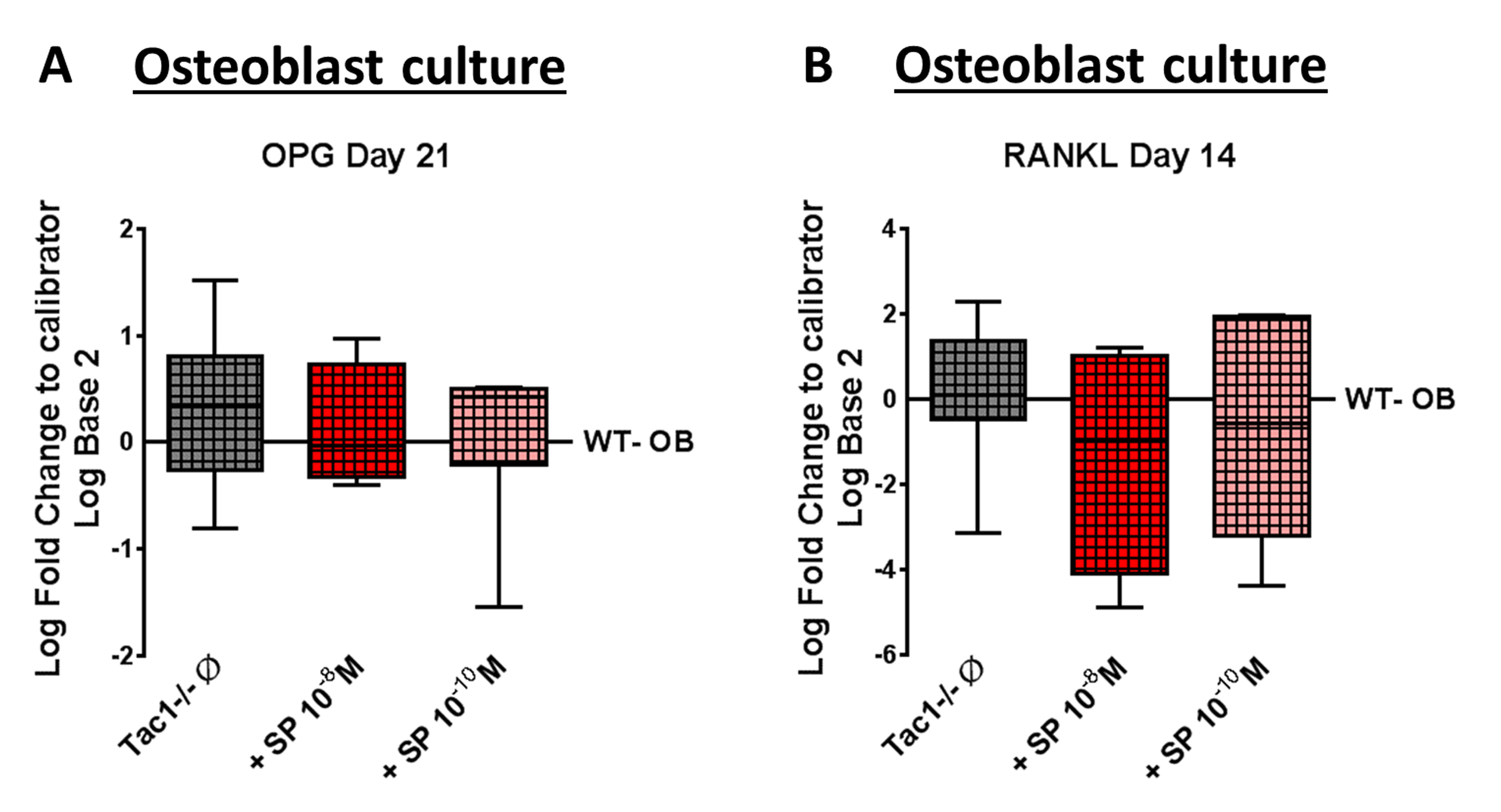

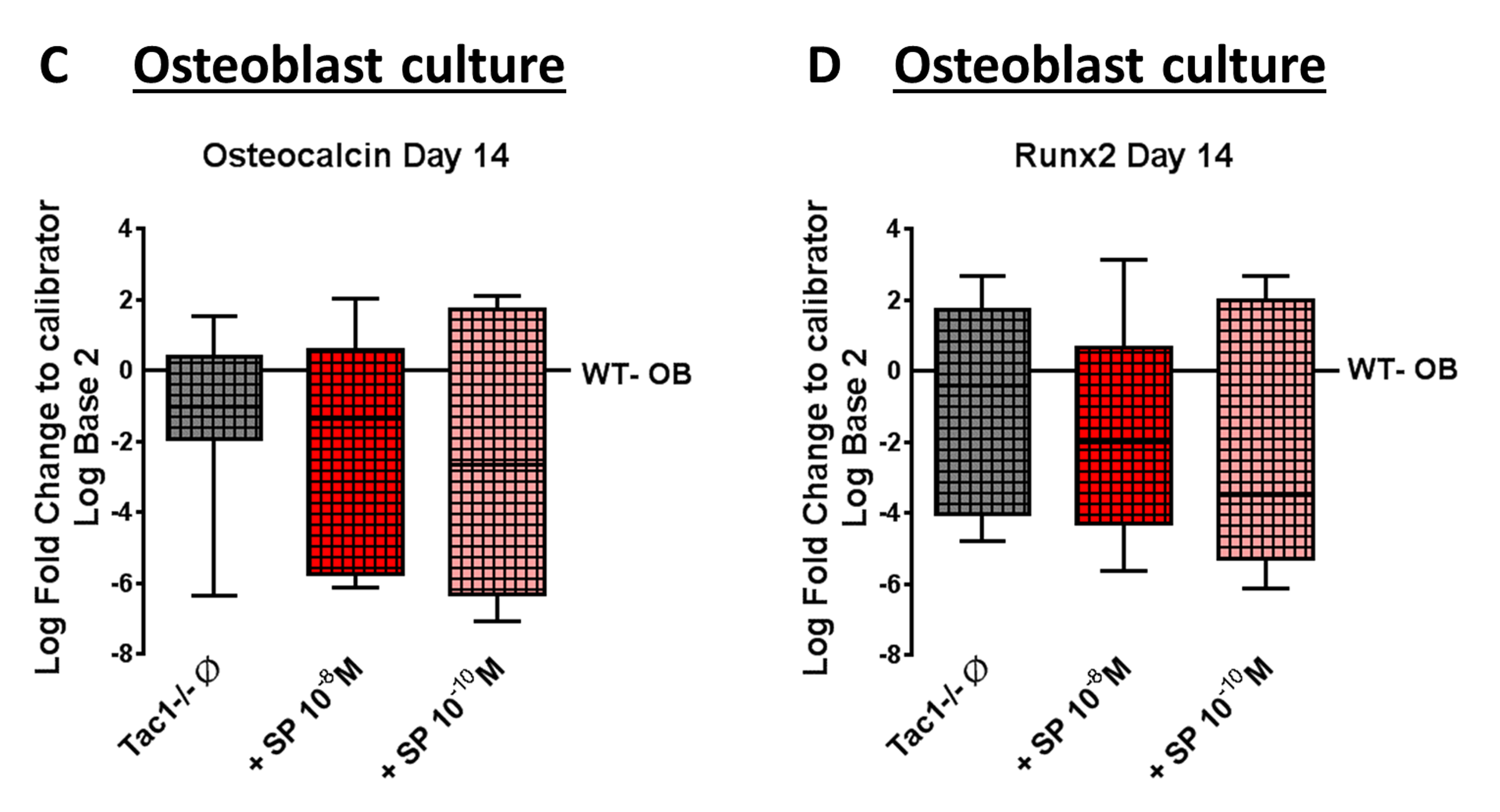


**Supplementary file 2: Osteoblast-specific marker gene expression**

Comparison of *TNFRSF11B* (OPG Day 21; A), *TNFSF11* (RANKL Day 14; B), *BGLAP* (osteocalcin day 14; C), *RUNX2* (Day 14; D) gene expression in Tac1-/- to WT osteoblasts after 14 (B-D) and 21 (A) days in osteogenic medium. N=8-16.

Results of RNA isolated from Tac1-/- cells were calibrated to RNA isolated from WT cells (= x-axes/0-line). Ø = no stimulation.


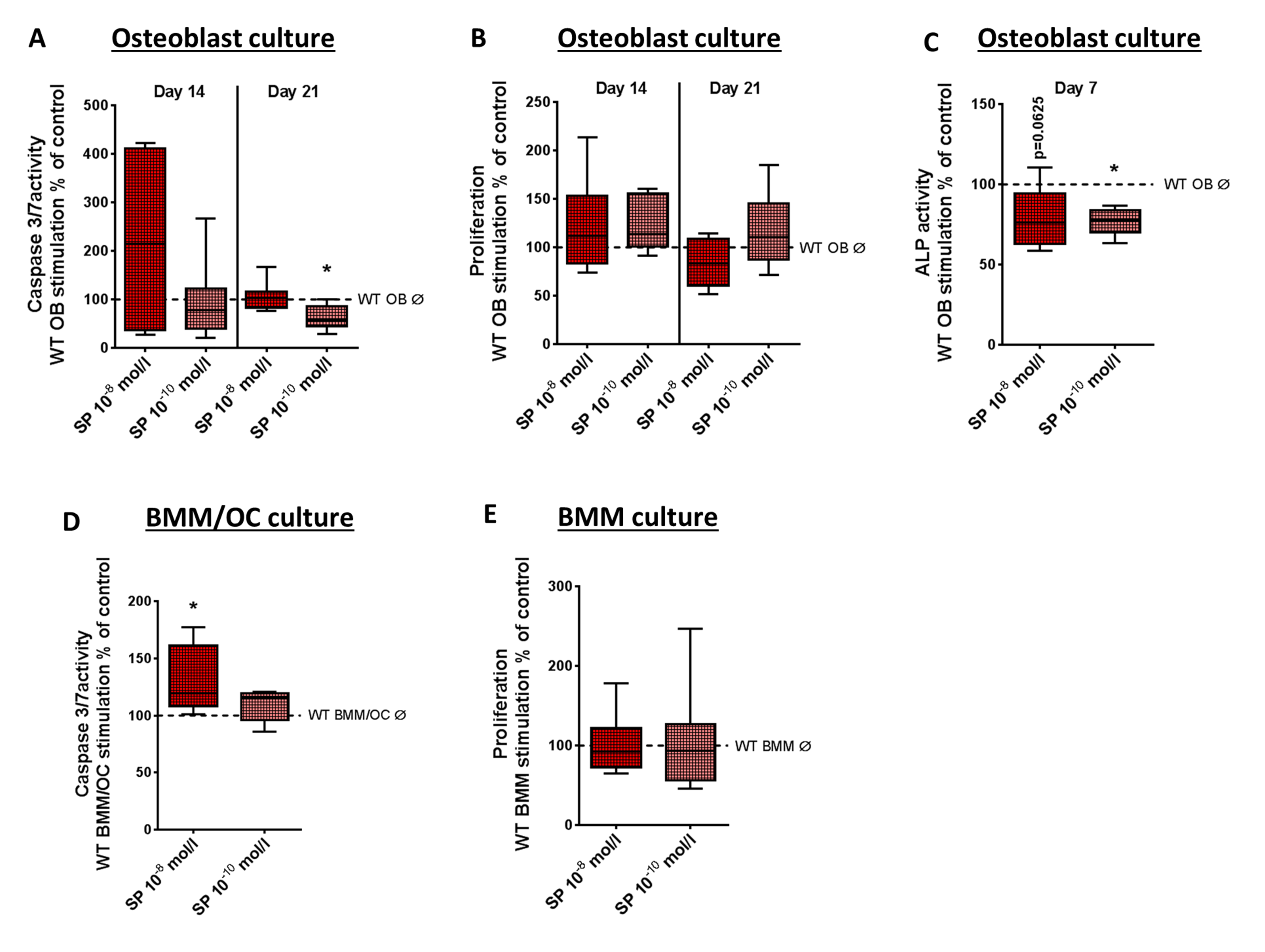


**Supplementary file 3: Apoptosis, proliferation rate and ALP activity of WT bone cells**

Caspase 3/7 activity (A) and proliferation (B) of WT osteoblasts after 14 and 21 days and ALP activity (C) of WT osteoblasts after 7 days culture in osteogenic medium. N=6-8

Caspase 3/7 activity (D) WT BMM/osteoclast cultures and proliferation (E) of BMM. N=6-7

BMM/osteoclast cultures and osteoblasts from WT mice were stimulated w/o SP 10^-8^/10^-10^ M for the last 24 h. Values of unstimulated (Ø = no stimulation) WT bone cells were set to 100%, results of stimulated WT bone cells were calibrated to unstimulated controls (dotted lines = 100%).

* = p ≤ 0.05 indicates difference to unstimulated WT cells.


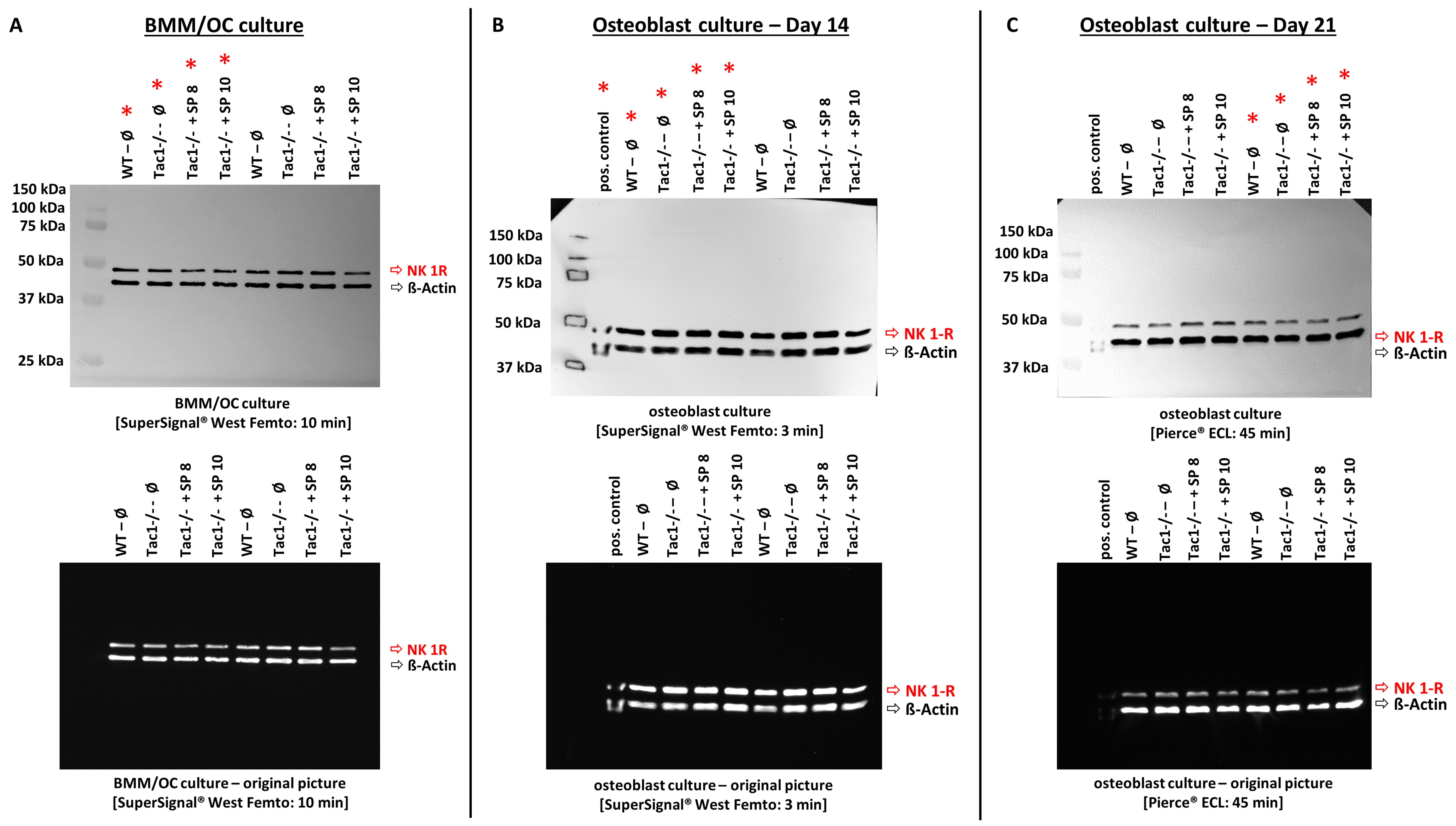


**Supplementary file 4: Full length and original western blot images**

(A) Full length (on top) and original (below) image of NK1R- and β-actin protein expression in WT and Tac1-/-BMM/osteoclast cell lysates , w/o stimulation with 10^-8^/10^-10^ M SP for 24 h. Lines with red asterisks are shown as representative image in Figure 2 E. Bands were detected with the SuperSignal® West Femto Maximum Sensitivity Substrate (Thermo Scientific, Rockford, USA). Exposure time is listed below the images. Ø = no stimulation. N=4-8

Full length (on top) and original (below) images of NK1R- and β-actin protein expression in WT and Tac1-/- osteoblast cell lysates after 14 (B) and 21 days (C) of osteogenic differentiation, w/o stimulation with 10^-8^/10^-10^ M SP for 24 h. Lines with red asterisks are shown as representative image in Figure 2 F. Bands were detected with the SuperSignal® West Femto Maximum Sensitivity Substrate (A) and the Pierce® ECL Western Blotting Substrate (B) (Thermo Scientific, Rockford, USA). Exposure time is listed below the images. N= 4-8
